# KAT5-mediated acetylation enhances the deubiquitination of HASPIN by OTUB2 and promotes breast cancer progression

**DOI:** 10.1038/s41419-026-08658-5

**Published:** 2026-03-27

**Authors:** Jiani Guo, Kang Kang, Shiqi Wang, Zhuqing Ji, Haoxuan Li, Yifan Zhu, Wei Song, Mingde Huang

**Affiliations:** 1 Department of Oncology, The Affiliated Huaian No.1 People’s Hospital of Nanjing Medical University, Huaian, China; 2 Department of Thyroid and Breast Surgery, The Affiliated Huaian No.1 People’s Hospital of Nanjing Medical University, Huaian, China

**Keywords:** Breast cancer, Post-translational modifications

## Abstract

Breast cancer (BC) remains the leading cause of global female cancer-related mortality, with poor survival in advanced stages driven largely by metastasis. Ubiquitination, a key post-translational modification, critically regulates the stability and function of various proteins, including oncoproteins and tumor suppressors, and deubiquitinases (DUBs) reversing this process are emerging therapeutic targets. In this study, we report that haploid germ cell-specific nuclear protein kinase (HASPIN) is highly expressed in BC and is closely associated with poor prognosis. We identify the DUB Otubain-2 (OTUB2) as a critical regulator of the oncogenic kinase HASPIN in BC. We demonstrate that OTUB2 binds to and deubiquitylates HASPIN, specifically counteracting its K48-linked polyubiquitination and subsequent proteasomal degradation. Acetylation of HASPIN at lysine 751 by acetyltransferase lysine acetyltransferase 5 (KAT5) enhances its affinity for OTUB2, promoting HASPIN stability. Functionally, OTUB2 depletion reduces HASPIN protein levels, while OTUB2 overexpression-induced HASPIN upregulation drives BC cell proliferation and invasion both in vivo and in vitro. These findings establish OTUB2 as a novel DUB for HASPIN and reveal a previously unknown regulatory axis involving KAT5, acetylation, OTUB2, ubiquitination, and HASPIN, which is crucial for BC progression. Consequently, HASPIN acts as an oncogene in BC and represents a promising new therapeutic target for intervention.

## Introduction

Breast cancer (BC) has been the leading cancer incidence and the second cancer-related death of females worldwide [1]. It is estimated about 420,000 new BC cases and 120,000 new BC deaths occurred in China each year [2]. With the development of diagnostic techniques and comprehensive treatments, recent years have witnessed the significant improvement of survival in early-stage BC patients. However, the 5-year survival rate of advanced BC is still at only 27% [3, 4]. Over 90% of cancer mortality is closely related to distant metastasis. Cancer cells negotiate a series of “metastatic cascade” with specific functions to form metastases, involving tumor heterogeneity, phenotypic plasticity, epithelial-mesenchymal transition (EMT), etc. [5] As key drivers of malignant biological behaviors, proteins remain the preferred anti-tumor therapeutic targets [6]. The ubiquitin-proteasome system is a ubiquitous type of post-translational modification (PTM) with great therapeutic potential. It promotes proteasomal degradation of proteins, thereby lowering their stability, while simultaneously altering interactions between distinct proteins. This ultimately results in precise modulation of protein functions. However, dysregulation of this system can also contribute to malignant development [7]. Besides, ubiquitination also controlled lysosomal degradation, protein trafficking and localization, as well as cellular signaling pathways [8].

Deubiquitinases (DUBs) specifically hydrolyze the isopeptide bond linking ubiquitin to substrate proteins, thereby reversing ubiquitination. DUBs play essential roles in modulating the intricate complexity of the ubiquitin system [9, 10]. Hitherto, researchers have discovered seven distinct DUB subfamilies, including ubiquitin-specific proteases, ovarian tumor proteases (OTUs), ubiquitin C-terminal hydrolases, Machado-Joseph disease proteases, motif-interacting with ubiquitin-containing novel DUB family (MINDYs), metalloprotease group or JAB1/MOV34/MPR1 (JAMMs), and zinc finger-containing ubiquitin peptidase 1 (ZUP1) [11–13]. By regulating the ubiquitin system, several DUBs have emerged as alternative and important therapeutic targets for cancers [14, 15]. Recently, a great number of studies concentrated on the significant role of OTUs family in numerous cellular processes, especially in cancers [16]. However, considering the complexity and diversity of the ubiquitin code, the biological activities and functions of DUBs are missing and need further clarification [9].

Constituting a major class of eukaryotic enzymes, protein kinases catalyze reactions critical to diverse cellular processes such as gene expression, membrane transport, proliferation, apoptosis, metabolism, and cell motility [17]. Haploid germ cell-specific nuclear protein kinase (HASPIN), also known as germ cell-specific gene 2 (GSG2), is an atypical serine/threonine protein kinase and a constitutively active enzyme, which is essential for mammalian cells’ proliferation and differentiation [18, 19]. It is reported that HASPIN contributes to chromosome segregation through phosphorylation of histone H3 at Thr3 during mitosis, and its dysregulation was observed in various cancers (such as pancreatic cancer [20], bladder cancer [21], cholangiocarcinoma [22], etc.), which exhibited its great potential in anticancer targeted therapy [19, 23, 24]. A recent study revealed the upregulation and biological function of HASPIN protein in BC cells, as well as its potential molecular mechanism in regulating cancer progression [25]. Nevertheless, the reason of its dysregulation, and the upstream regulation mechanism of HASPIN protein, especially the involvement of DUBs, are still not clear.

In this study, we found that DUB Otubain-2 (OTUB2) prevented the ubiquitination and degradation of HASPIN protein in BC. Depletion of OTUB2 increased the abundance of K48-linked polyubiquitinated HASPIN. Moreover, acetylation of HASPIN at a single residue, lysine 751 (K751), by KAT5 showed markedly enhanced affinity for OTUB2 binding with and promoting stability of HASPIN protein. Our investigation established the OTUB2-HASPIN axis as a novel oncogenic driver in BC. Overexpression of either OTUB2 or HASPIN enhanced tumor cell proliferation and invasion, culminating in accelerated growth and metastasis of xenografts. Furthermore, we characterized OTUB2, an OTU family deubiquitinase, as the specific DUB for HASPIN and a promising therapeutic target in BC.

## Materials and methods

### Patients and materials

Twenty-eight paired BC and adjacent tissue samples were obtained from The Affiliated Huaian No.1 People’s Hospital of Nanjing Medical University during June 2019 to December 2020. All patients underwent surgical resection and had received no neoadjuvant radiotherapy or chemotherapy. This study was approved by the ethics committee of The Affiliated Huaian No.1 People’s Hospital, Nanjing Medical University (KY-2024-299-01). A tissue microarray comprising 130 cases of BC tissues was purchased from Shanghai Xinchao Biotechnology (Shanghai, China).

### Immunohistochemistry (IHC)

Protein levels were examined in the tissue microarray by immunohistochemistry. The tissue slides were baked at 60 °C for 2 h, deparaffinized by dimethylbenzene and hydrated by ethanol. We used citrate buffer to repair antigens, 3% H_2_O_2_ to block endogenous peroxidase, and bovine serum albumin to avoid non-specific binding. The slides then went through primary antibody incubation at 4 °C overnight, and added with secondary antibodies. Next, we stained the samples with dimethylbenzene and hematoxylin, sealed the slides with neutral gum, and scanned the tissues under fluorescent microscope. Protein expressions were evaluated by two independent observers, and scored by multiplying the intensity and percentage scores.

### Cell culture

The three BC cell lines (MCF7, MDA-MB-231, BT549) and human HEK-293T cells were obtained from Cell Bank of the Chinese Academy of Sciences (Shanghai, China), authenticated by STR. Cells were regularly tested for mycoplasma contamination using the PCR-based mycoplasma screening Kit (Sigma-Aldrich, Missouri, USA) every 3 months, and cultured in DMEM/RPMI-1640 with 10% fetal bovine serum (FBS, Clark Bioscience, Virginia, USA) and 1% penicillin-streptomycin at 37 °C/5% CO₂.

### Plasmids

The HA-OTUB2 plasmid was generated by subcloning human OTUB2 cDNA into the pcDNA3.1-HA vector. Similarly, Myc-HASPIN was constructed through cloning HASPIN cDNA into pcDNA3.1-Myc. For lentiviral expression, OTUB2 cDNA was inserted into pLVX-Puro-HA to create HA-tagged lentiviral vectors, whereas HASPIN was engineered into pLVX-Puro-Myc for Myc-tagged lentiviral production. GST-tagged OTUB2 and its C51S mutant were expressed using the pGEX-6P-1 vector. HASPIN truncation mutants (HASPIN-1/2/3) and site mutants (K462, K470, K483, K584, K618, K664, K768) were subcloned into pcDNA3.1-Myc. Site-directed mutagenesis was performed using Myc-HASPIN as template to generate K751R/Q mutants, and HA-OTUB2 as template for C51S mutants. Ubi-WT and Ubi mutants (K6, K11, K27, K29, K33, K48, K63, K48R) were cloned into the pCDNA3.1-His-C vector. Human acetyltransferase cDNAs were cloned into pcDNA3.1-Flag vector to generate Flag-KAT2A, Flag-KAT2B, Flag-KAT5, Flag-CBP, and Flag-P300 plasmids. We used GV248 lentiviral vectors to express OTUB2 or HASPIN shRNAs. The information of shRNAs is listed in Supplementary Table S1.

Plasmids were transfected into cells with Lipofectamine 3000 (Thermo Fisher, Massachusetts, USA). 48 h after transfection, cells were used for further experiments. For lentiviral infection, cells were infected with constructed viruses, and then went through puromycin selection to establish stable cell lines. Transductions were performed using a standardized MOI, as determined by prior titration.

### Antibodies and reagents

The antibodies and reagents used in this study are provided in Supplementary Table S2.

### Transwell assay

The transfected BC cells were harvested and resuspended in serum-free culture medium to suitable density, added to the upper chambers pre-added with matrix gel (BD Biosciences, California, USA) on their bottom surfaces. The upper chambers with 200 μl suspended cells were then placed into the lower chambers containing 600 µL medium with 15% FBS for 24 h, and non-migratory cells on the upper membrane surface were removed. The fixed chamber membranes (4% paraformaldehyde) were then stained with 0.1% crystal violet and imaged microscopically.

### Wound-healing assay

The transfected cells were pre-seeded in 6-well plates with 100% confluent in serum-free media for 24 h. The cell monolayers were scratched by a sterile 200 μl pipette tip to create a straight wound in the middle of the wells. We took images of the wound at 0 h, 48 h after scratching, and calculated the remaining areas without cells.

### Colony formation assays

Transfected BC cells were digested by trypsin and resuspended in complete medium at 1 × 10⁶ cells/ml. Appropriate dilutions yielded 500–1000 cells per well in 6-well plates. Following 14-day incubation (37 °C, 5% CO₂), colonies were quantified after 0.1% crystal violet staining.

### In vivo xenograft model of mice

Nude mice (BALB/c, female, 6 weeks old, 18–22 g, each group was randomly assigned to six mice) were purchased from the Beijing Vital River Laboratory Animal Technology Co., Ltd (Beijing, China), and housed within specific pathogen-free (SPF) animal facilities. The transfected MDA-MB-231 cells (1 × 10^7^/ml) were suspended in 200 μl PBS and subcutaneously injected into the armpits of mice to access the tumor formation ability, or into the tail vein of mice to evaluate the tumor metastasis ability. The tumor volumes were monitored every 3 days. At the indicated endpoints, mice were euthanized, and tumors were completely resected. The tumors were sectioned, measured, weighed, and stained with Hematoxylin-eosin (H&E) to confirm BC-derived tumor. The study was approved by the Ethics committee of Huaian No. 1 People’s Hospital (DW-P-2024-029-01) and was conducted following the National Animal Care and Ethics Institution guidelines.

### Western blotting (WB)

Cell/tissue lysates were prepared using RIPA buffer supplemented with protease/phosphatase inhibitors (Sigma-Aldrich, USA), followed by ice-cold sonication. Proteins were resolved by SDS-PAGE, transferred to 0.45 μm PVDF membranes (Millipore, Massachusetts, USA), blocked with 5% non-fat milk in TBST, and incubated overnight at 4 °C with primary antibodies. The membranes were then added with secondary antibodies and room temperature, and subjected to enhanced chemiluminescence (ECL, NCM Biotech, China).

### Immunofluorescence (IF)

Cultured BC cells were sequentially processed as follows: PBS wash, 4% paraformaldehyde fixation, 0.1% Triton X-100 permeabilization, and 3% BSA blocking, followed by overnight incubation with primary antibodies at 4 °C. Then the cells were added with fluorescent secondary antibodies at room temperature, stained with DAPI (Sigma-Aldrich, USA) for nuclei visualization, and photographed under a laser scanning confocal microscope.

### Immunoprecipitation (IP)

The transfected cells were harvested and lysed with the specific lysis buffer. The Protein A/G Magnetic Beads (MCE, New Jersey, USA) were washed and incubated with pre-diluted antibodies at 4 °C for 2 h. Then the antibody-bead complex was added into the cell lysate at 4 °C for 2 h. The beads were isolated by a magnetic frame, and the antigen-antibody complex boiled with SDS loading buffer, which were subjected to further immunoblotting.

### LC-MS/MS analysis

HASPIN was isolated from MCF7 cell lysates via protein A/G magnetic immunoprecipitation. Immunoprecipitated proteins were electrophoresed and Coomassie blue-stained. Target bands were excised for in-gel tryptic digestion, dried, and analyzed by mass spectrometry to identify protein components.

### GST pull-down assay

The Myc-HASPIN protein was purified from HEK-293T cells. The recombinant GST-tag or GST-tagged proteins were purified from *E. coli*, and bound to glutathione-Sepharose resin with Myc- HASPIN at 4 °C for 5 h. The complex was then washed with GST buffer and boiled with SDS loading buffer for further immunoblotting.

### Proximity ligation assay (PLA)

Fixed BC cells (4% paraformaldehyde) were blocked (5% BSA, 1 h), incubated with primary antibodies (4 °C, overnight), and subjected to Duolink® In Situ Proximity Ligation Assay kit (PLA, Sigma-Aldrich, USA) according to kit protocol. DAPI counterstaining preceded confocal imaging.

### In vivo and vitro deubiquitination assays

For the in vivo deubiquitination assays, cells transfected with His-Ubi were pre-treated with 10 μM MG-132 for 6 h, and lysed with RIPA buffer containing 1% SDS. The lysis was then dilute to 0.2% SDS, added with the HASPIN antibody for immunoprecipitation, and ubiquitination level was further analyzed through Western blot. For the in vitro deubiquitination assays, His-Ubi and Myc-HASPIN were co-transfected in HEK-293T cells for 48 h. The cells went through immunoprecipitation with anti-Myc affinity magnetic beads to enrich HASPIN protein. HA-tagged OTUB2 and its C51S mutant were isolated from HEK-293T cells using anti-HA magnetic beads. After incubation in deubiquitylation buffer (50 mM Tris-HCl pH 7.4, 1 mM DTT, 1 mM MgCl₂) at 37 °C for 2 h, ubiquitination levels were assessed by Western blotting.

### Statistical analysis

All experiments were independently conducted at least 3 times, presented as mean ± SD, and data analysis was performed on the SPSS 26.0 software package or GraphPad Prism 9 system. The comparison of means between two groups covered Chi-squared and unpaired two-tailed Student’s *t* test, as well as one-way ANOVA-post-hoc pairwise analysis to compare difference among multiple groups. Patient survival differences were evaluated by Kaplan-Meier analysis, while gene correlations were assessed using chi-square tests and linear regression. *P* < 0.05 represented significant differences.

## Results

### The elevated protein levels of HASPIN in BC were correlated with higher tumor malignancy and poorer patient prognosis

**Fig. 1:**
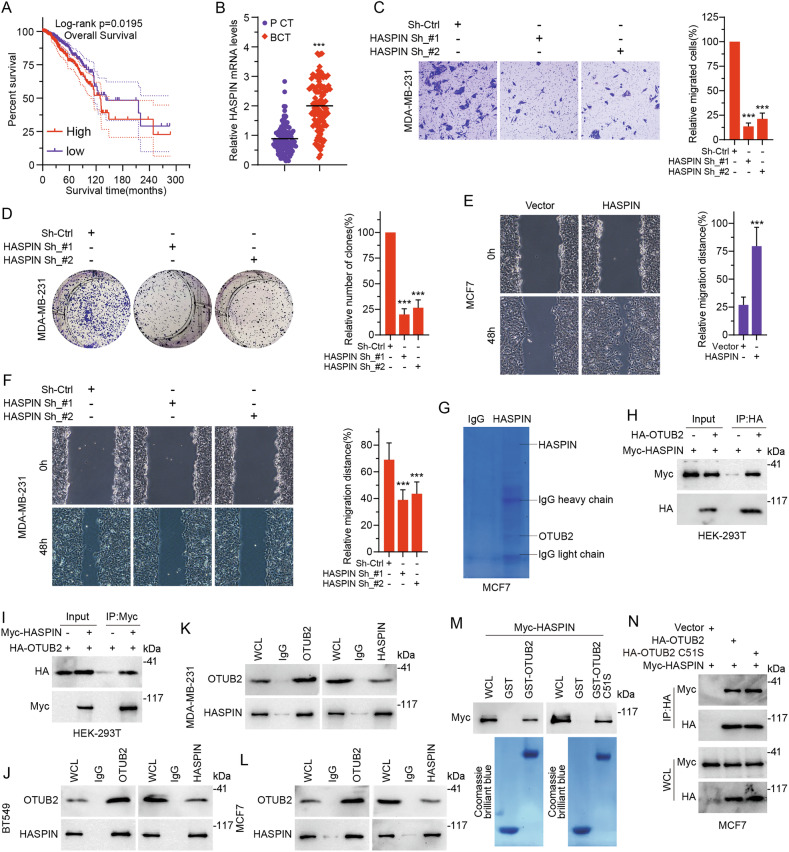
Investigation of HASPIN expression, function, and its interaction with OTUB2 in breast cancer. **A** BC patients with high or low HASPIN expression showed different overall survival analyzed by Kaplan-Meier test. **B** Relative HASPIN mRNA levels varied in paired control tissues (P CT, *n* = 113) and breast cancer tissues (BCT, *n* = 113) from TCGA-BC database. **C** Transwell assay was performed in MDA-MB-231 cells with HASPIN knockdown and control, showing representative images of migrated cells on the left and relative invasive cell counts on the right. **D** Colony formation assay was performed in MDA-MB-231 cells with HASPIN knockdown and control, presenting representative images of colonies on the left and relative numbers of colonies on the right. **E** Wound healing assay was conducted in MCF7 cells with HASPIN overexpression (Vector vs. HASPIN), showing representative images at 0 h and 48 h on the left and relative migration distances on the right. **F** Wound healing assay was conducted in MDA-MB-231 cells with HASPIN knockdown and control, showing representative images at 0 h and 48 h on the left and relative migration distances on the right. **G** Immunoprecipitation (IP) was carried out with anti-HASPIN or control IgG antibody followed by Coomassie Brilliant Blue in MCF7 cells. **H** Co-IP was carried out with anti-HA antibody to detect the interaction between HA-OTUB2 and Myc-HASPIN in HEK293T cells. **I** Co-IP was carried out with anti-Myc antibody to detect the exogenous interaction between HA-OTUB2 and Myc-HASPIN in HEK293T cells. **J**, **K**, **L** Co-IP confirms endogenous interaction between HASPIN and OTUB2 proteins in BC cells. **M** GST pull-down assay used Myc-HASPIN to pull down GST-OTUB2 or GST-OTUB2 C51S, shown as a protein-gel-like image. **N** Immunoblotting after Co-IP with anti-HA antibody was conducted to detect the interaction between HA-OTUB2 (wild-type or C51S mutant) and Myc-HASPIN in MCF7 cells. ***p < 0.001.

To explore the expression pattern and clinical significance of HASPIN in BC patients, we analyzed HASPIN mRNA expression in BC tissues derived from TCGA-BC database, which showed that HASPIN mRNA expression levels were significantly higher in BC tissues than both in paired or unpaired control tissues. (Fig. 1B and Supplementary Fig. S1A). Survival analysis indicated that BC patients with high HASPIN expression were related with poor overall survival (Fig. 1A). To clarity the biological function of HASPIN in BC progression, we transfected BC cell lines with plasmids to constructed different HASPIN expression patterns. The transwell assays indicated that invasion ability of BC cells was suppressed following HASPIN inhibition (Fig. 1C), as well as promoted after HASPIN overexpression (Supplementary Fig. S1B). The colony formation assays showed that proliferation activity of BC cells was inhibited with HASPIN knockdown (Fig. 1D), and HASPIN upregulation could also promote BC cell proliferation (Supplementary Fig. S1C). Moreover, HASPIN could enhance the migration ability of BC cells in vitro (Fig. 1E, F). Collectively, our findings demonstrated that HASPIN was frequently overexpressed in BC tissues and that its high expression was associated with poor patient prognosis. Functionally, HASPIN promoted the malignant phenotype of BC cells by enhancing their proliferation, migration, and invasive capabilities in vitro. These results supported the hypothesis that HASPIN played a critical oncogenic role in BC progression and suggested its potential as a prognostic biomarker and therapeutic target.

### OTUB2 interacts with HASPIN

Since the tumor-promoting activity of HASPIN in BC development was observed, we wondered the underlying mechanism in which HASPIN was upregulated. The LC-MS/MS analysis identified a DUB OTUB2 as a possible HASPIN-binding protein in MCF7 cells (Fig. 1G). We then performed Co-Immunoprecipitation (Co-IP) to confirm their interaction in HA-tagged OTUB2 and Myc-tagged HASPIN co-transfected HEK293T cells (Fig. 1H, I). Moreover, the endogenous OTUB2-HASPIN interactions were also detected in MDA-MB-231, BT549 and MCF7 cells (Fig. 1J–L). The GST pull-down assay showed that purified GST-tagged OTUB2 could bind to Myc-tagged HASPIN under cell-free conditions (Fig. 1M), suggesting a direct combination between OTUB2 and HASPIN. Then we performed Co-IP assays to detect the Myc-tagged HASPIN in immunoprecipitates of either HA-tagged OTUB2 or HA-tagged OTUB2 C51S [inactivation mutation, cysteine(C) to serine(S)] in MCF7 and HEK-293T cells, which demonstrated that both OTUB2 with or without DUB activity could bind with HASPIN (Fig. 1N and Supplementary Fig. S1D). Next, we used immunofluorescence (IF) staining to confirm the colocalization of OTUB2 and HASPIN protein in the nucleus of BT549, MDA-MB-231 and MCF7 cells (Supplementary Fig. S1E). Concurrently, through the meticulous detection of fluorescence intensity along a specific distance, the co-distribution patterns of OTUB2 and HASPIN signals within these three cell lines were elaborately revealed (Supplementary Fig. S1F–H). Additionally, the endogenous interaction between HASPIN and OTUB2 was further validated by in-situ proximity ligation assay (PLA) in BC cells (Supplementary Fig. S1I). To clarify the binding domain of the two proteins, we expressed three truncated mutants (Myc-HASPIN-1, Myc-HASPIN-2 and Myc-HASPIN-3), which mapped the specific regions according to its functional features, and proved the protein kinase domain of HASPIN was essential for their interaction in HEK293T cells (Supplementary Fig. S1J–L). Furthermore, we used the PhosphoSitePlus® PTM database to predict the potential specific lysine (K) residues on HASPIN that showed enriched ubiquitination, including K462, K470, K483, K584, K618, K664, K768 in the kinase domain. Based on the prediction results, we generated HASPIN mutants where the identified key lysine residues were substituted with arginine (K-to-R mutants). Subsequent ubiquitination assays demonstrated that mutation of the specific sites (K618) significantly attenuated the polyubiquitination of HASPIN (Supplementary Fig. S2A–C). Overall, our results suggested that OTUB2 could directly bind with HASPIN protein in BC.

### OTUB2 maintains HASPIN stability

**Fig. 2:**
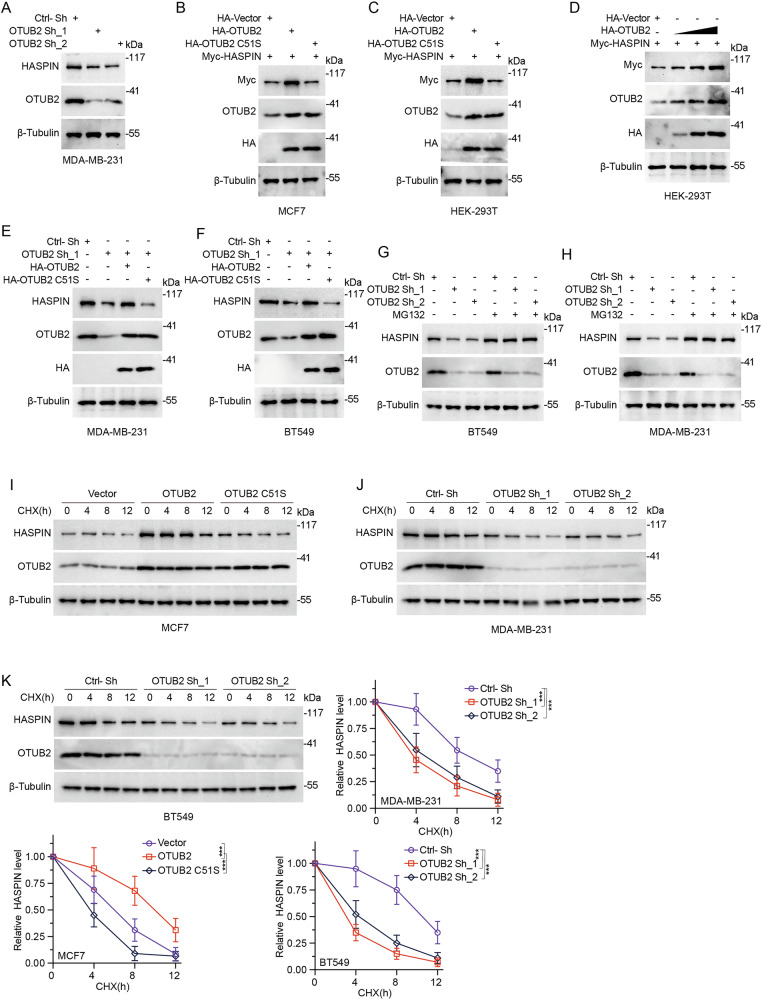
OTUB2 maintains HASPIN stability. **A** Western blot showed HASPIN and OTUB2 protein levels in MDA-MB-231 cells transfected with control or OTUB2 shRNA plasmids. **B**, **C** Overexpression of OTUB2, but not OTUB2 C51S, resulted in elevated HASPIN protein level in MCF7 and HEK-293T cells. **D** Western blot showed effect of different doses of OTUB2 overexpression on HASPIN protein levels in HEK-293T cells. **E**, **F** Western blot indicated that HASPIN protein downregulation followed by OTUB2 knockdown could be reversed by OTUB2 overexpression, but not OTUB2 C51S, in MDA-MB-231 and BT549 cells. **G**, **H** Western blot revealed that HASPIN protein downregulation followed by OTUB2 knockdown could be reversed by MG132 (proteasome inhibitor) treatment in MDA-MB-231 and BT549 cells. **I** Western blot showed the time-course of HASPIN and OTUB2 levels in MCF7 cells after CHX (protein synthesis inhibitor) treatment with different OTUB2 manipulations. **J**, **K** Western blot showed the time-course of HASPIN and OTUB2 levels in MDA-MB-231 and BT549 cells after CHX treatment with control or OTUB2 shRNA plasmids. ***p < 0.001.

On the basic of OTUB2-HASPIN interaction, we wonder whether the DUB OTUB2 could regulate HASPIN protein degradation and stabilization. Firstly, we applied specific shRNA plasmids for OTUB2 knockdown in BC cells (Supplementary Fig. S2D, E), which indicated that depletion of OTUB2 caused a decreased expression of HASPIN protein (Fig. 2A and Supplementary Fig. S2G). Besides, we overexpressed HASPIN protein with HA-OTUB2 or HA-OTUB2 C51S (Supplementary Fig. S2F), which revealed that OTUB2 without enzymatic activity could not increase HASPIN protein expression in MCF7 and HEK293T cells (Fig. 2B, C). We also found that OTUB2 could enhance the protein level of HASPIN in a dose-dependent manner (Fig. 2D and Supplementary Fig. S2H). The decreased HASPIN expression due to OTUB2 knockdown was reversed by HA-OTUB2 transfection, but not HA-OTUB2 C51S, indicating that the enzymatic activity of OTUB2 was essential for HASPIN protein stabilization (Fig. 2E, F). Additionally, we treated OTUB2-deficient cells with MG132 (proteasome inhibitor) or Chloroquine (CQ, lysosome inhibitor) to further specify the molecular mechanism of OTUB2-mediated HASPIN stabilization, which found that OTUB2 knockdown could still diminish HASPIN protein expression in BC cells added with CQ, but this effect was abolished by MG132 treatment, demonstrating that OTUB2 stabilized HASPIN protein by the ubiquitin-proteasome pathway (Fig. 2G, H and Supplementary Fig. S2I, J). We then added protein synthesis inhibitor cycloheximide (CHX) in OTUB2-deficient or OTUB2-overexpressed BC cells, which found that overexpressed OTUB2 led to a remarkable improvement of endogenous HASPIN protein stability, and OTUB2 depletion could result in HASPIN destabilization (Fig. 2I–K). To sum up, our results suggested that OTUB2 contributed to HASPIN protein degradation and stabilization.

### OTUB2 deubiquitinates HASPIN

**Fig. 3:**
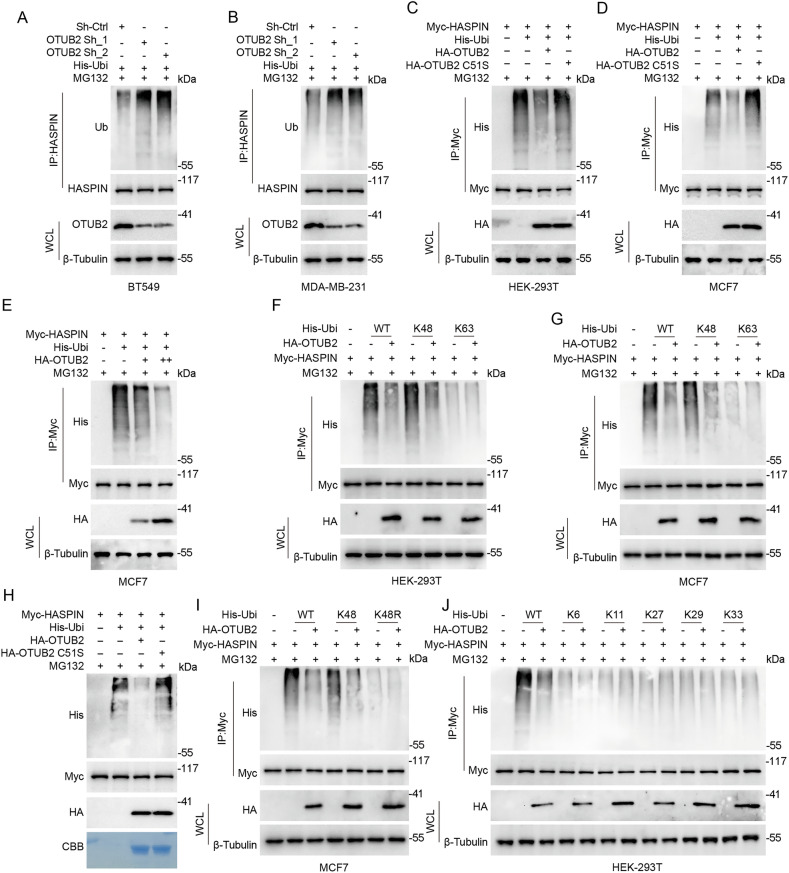
OTUB2 deubiquitinates HASPIN. **A**, **B** Knockdown of OTUB2 resulted in elevated ubiquitination of HASPIN in BT549 and MDA-MB-231 cells co-transfected with OTUB2 shRNA plasmids (OTUB2 Sh_1, OTUB2 Sh_2), His-Ubi, and treated with MG132. **C**, **D** Co-IP showed that OTUB2, but not OTUB2 C51S, could induce the deubiquitination of HASPIN protein in HEK-293T and MCF 7 cells co-transfected with Myc-HASPIN, His-Ubiquitin (His-Ubi), HA-OTUB2 or HA-OTUB2 C51S, and treated with MG132. **E** Co-IP showed that OTUB2 could induce the deubiquitination of HASPIN protein in a dose-dependent manner in MCF7 cells co-transfected with different doses of HA-OTUB2, Myc-HASPIN, His-Ubi and treated with MG132. **F**, **G** Co-IP demonstrated that OTUB2 could induce the K48-linked deubiquitination of HASPIN protein in HEK-293T and MCF7 cells co-transfected with Myc-HASPIN, His-Ubi (wild-type, K48 or K63 mutant), HA-OTUB2, and treated with MG132. **H** In vitro deubiquitination assay indicated that OTUB2, but not OTUB2 C51S, could induce the deubiquitination of HASPIN protein in the presence of Myc-HASPIN, His-Ubi, HA-OTUB2 or HA-OTUB2 C51S, and MG132, with Coomassie Brilliant Blue staining as a loading control. **I** Co-IP revealed that OTUB2 could not induce the deubiquitination of HASPIN protein in MCF7 cells co-transfected with Myc-HASPIN, His-Ubi K48R mutant, HA-OTUB2, and treated with MG132. **J** Co-IP revealed that OTUB2 could not induce the deubiquitination of HASPIN protein in HEK-293T cells co-transfected with Myc-HASPIN, His-Ubi mutants (K6, K11, K27, K29, K33), HA-OTUB2, and treated with MG132.

Since OTUB2 usually acts as a DUB, we wonder whether OTUB2 could deubiquitinate HASPIN in BC. The deubiquitination assay revealed that the polyubiquitination level of HASPIN increased remarkably after OTUB2 knockdown in BT549 and MDA-MB-231 cells (Fig. 3A, B). Additionally, HA-OTUB2 transfection obviously attenuated the polyubiquitination level of HASPIN, but HA-OTUB2 C51S had no effect (Fig. 3C, D). We also found that OTUB2 could reduce the polyubiquitination level of HASPIN in a dose-dependent manner (Fig. 3E and Supplementary Fig. S3A). Then we co-transfected HA-OTUB2, Myc-HASPIN, and His-tagged ubiquitin plasmids with distinct Ub chain linkages (K6, K11, K27, K29, K33, K48, K63) in HEK-293T and MCF7 cells to determine the ubiquitin chain regulated by OTUB2, which demonstrated that the K48-linked polyubiquitin chain of HASPIN was significantly affected by OTUB2 overexpression (Fig. 3F, G, J and Supplementary Fig. S3C). The ubiquitination level of HASPIN showed no difference in cells transfected with Ub mutant K48R [inactivation mutation, lysine(K) to arginine(R)] plasmid (Fig. 3I and Supplementary Fig. S3B). The incubation of polyubiquitinated HASPIN and purified HA-OTUB2 or HA-OTUB2 C51S in cell-free conditions showed that only the OTUB2 wild type group could bind with and disassemble the ubiquitin moieties of HASPIN in vitro, which suggested that HASPIN was a direct deubiquitinated substrate of OTUB2 (Fig. 3H). In summary, OTUB2 could enhance HASPIN stability by selectively removing K48-linked polyubiquitination chains from the HASPIN protein.

### OTUB2 promotes HASPIN-mediated proliferation and metastasis in BC

**Fig. 4:**
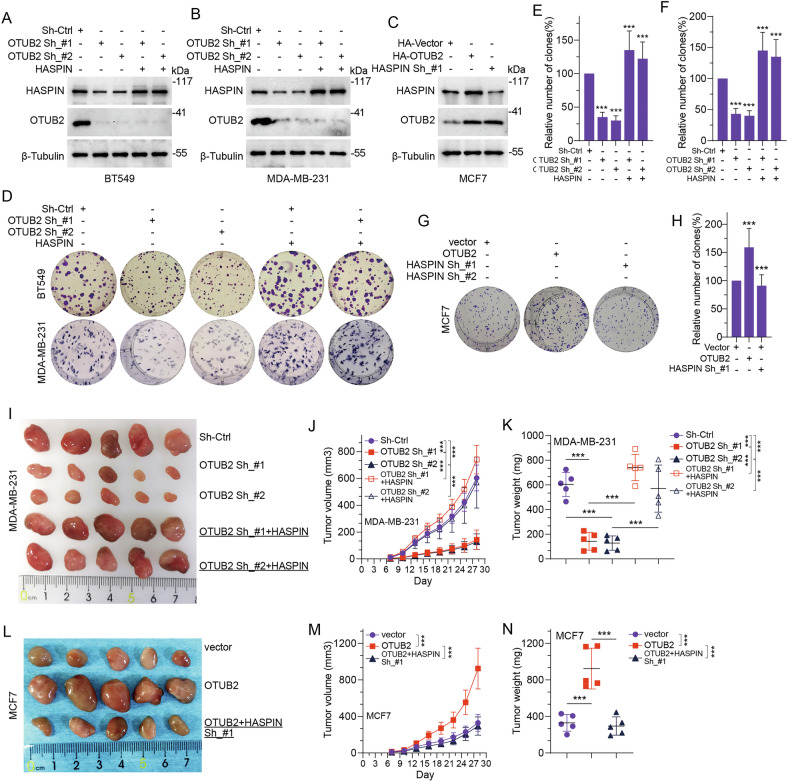
OTUB2 promotes HASPIN-mediated proliferation in BC. **A**–**C** Protein levels of HASPIN, OTUB2, and β-Tubulin (loading control) in different breast cancer cell lines (BT549, MDA-MB-231, MCF7) were detected by Western blot after transfection with OTUB2 shRNA plasmids (OTUB2 Sh_#1, OTUB2 Sh_#2) and HASPIN overexpression plasmids (HASPIN), or OTUB2 overexpression plasmids (HA-OTUB2), and HASPIN shRNA plasmid (HASPIN Sh_#1). **D**–**F** Results of colony formation assay showed the relative number of colonies (compared to the control group) of breast cancer cells (BT549, and MDA-MB-231) in different treatment groups (OTUB2 Sh_#1, OTUB2 Sh_#2, and HASPIN plasmids). **G**, **H** Results of colony formation assay showed the relative number of colonies (compared to the control group) of MCF cells in different treatment groups (OTUB2, HASPIN Sh_#1, and HASPIN Sh_#2 plasmids). **I**–**K** Representative images, volume time curves and volume weight changes showed the subcutaneous tumors excised from nude mice formed by MDA-MB-231 cells in different treatment groups (Sh-Ctrl, OTUB2 Sh_#1, OTUB2 Sh_#2, OTUB2 Sh_#1 + HASPIN, and OTUB2 Sh_#2 + HASPIN). **L**–**N** Representative images, volume time curves and volume weight changes showed the subcutaneous tumors excised from nude mice formed by MCF7 cells in different treatment groups (vector, OTUB2, and OTUB2 + HASPIN sh_#1). ***p < 0.001.

**Fig. 5:**
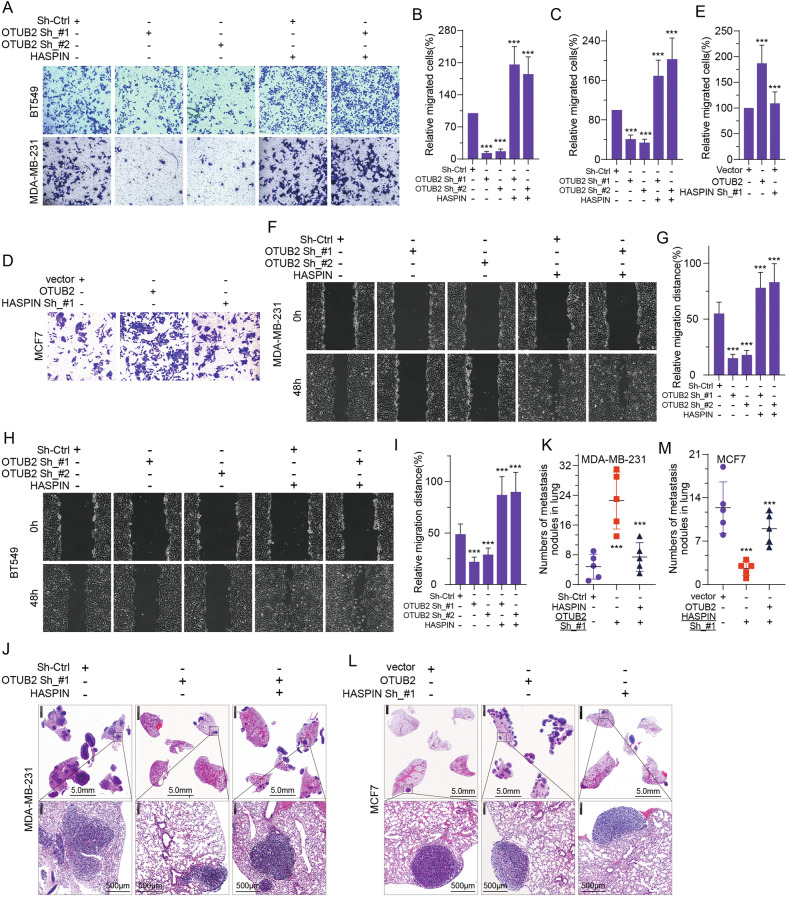
OTUB2 promotes HASPIN-mediated metastasis in BC. **A**–**C** Representative images and graphic representation of stained migrated cells of BT549 and MDA-MB-231 cell lines in different treatment groups (Sh-Ctrl, OTUB2 Sh_#1, OTUB2 Sh_#2, and HASPIN plasmids). **D**, **E** Representative images and graphic representation of stained migrated cells of MCF cell line in different treatment groups (vector, OTUB2, and HASPIN Sh_#1 plasmids). **F**–**I** Representative images and graphic representation of wound healing assays at 0 h and 48 h for different breast-cancer cell lines (MDA-MB-231, BT549) transfected with Sh-Ctrl, OTUB2 Sh_#1, OTUB2 Sh_#2, and HASPIN plasmids. **J**, **K** Representative H&E stained images and graphic representation of lung metastatic nodules derived from MDA-MB-231 cells transfected with Sh-Ctrl, OTUB2 Sh_#1, and HASPIN plasmids. **L**, **M** Representative H&E stained images and graphic representation of lung metastatic nodules derived from MCF7 cells transfected with vector, OTUB2, and HASPIN Sh_#1 plasmids. Scale bar, 5 mm (upper) and 500 μm (lower). ***p < 0.001.

To assess the effect of OTUB2-mediated deubiquitination of HASPIN on cell malignant phenotypes in BC, we firstly mediated OTUB2 protein expression level using specific OTUB2 shRNA or HA-OTUB2 plasmids in different BC cell lines. The results indicated that OTUB2 knockdown led to prominently repression of HASPIN protein level in vitro, which could be reversed by HASPIN overexpression (Fig. 4A, B). On the contrary, overexpression of OTUB2 resulted in elevated HASPIN protein level, which could be rescued by HASPIN knockdown (Fig. 4C). Next, we applied colony formation assay, transwell assay and wound-healing assay in vitro, as well as subcutaneous xenograft model and lung metastasis model in vivo, to evaluate the effect of OTUB2-mediated deubiquitination of HASPIN on cell proliferation, migration and metastasis. The results revealed that downregulation of OTUB2 led to inhibition of cell malignant phenotypes, which could be reversed by HASPIN overexpression (Figs. 4D–F, 5A–C, F–I). Conversely, overexpression of OTUB2 resulted in enhanced cell malignancy in BC, which could also be impaired by HASPIN repression (Figs. 4G, H, 5D, E and Supplementary Fig. S4A, B). The xenograft model showed consistent trends in vivo, where OTUB2 knockdown impeded the tumor progression (Figs. 4I–K, 5J, 5K), and OTUB2 upregulation resulted in promotion in tumor malignant behavior (Figs. 4L–N, 5L, M). Similarly, both outcomes could be reversed by upregulation or downregulation of HASPIN, respectively. In conclusion, our findings suggested that OTUB2 could promote BC proliferation and metastasis through stabilization of HASPIN both in vitro and in vivo.

### KAT5-mediated acetylation of HASPIN at K751 increases its interaction with OTUB2

**Fig. 6:**
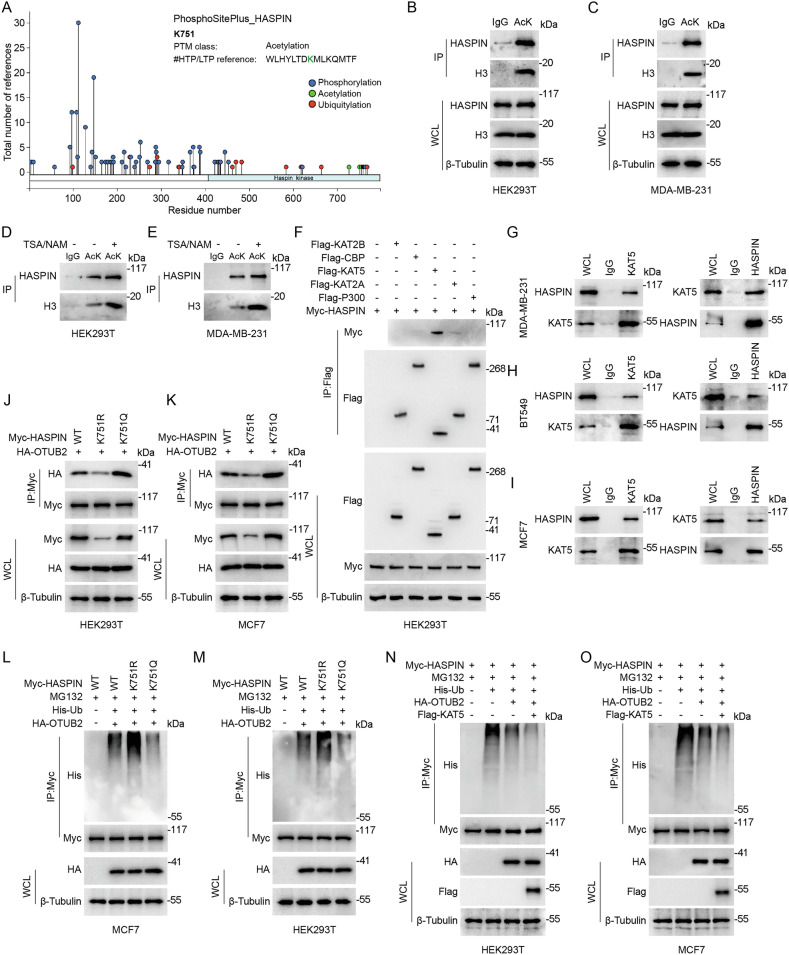
KAT5-mediated acetylation of HASPIN at K751 increases its interaction with OTUB2. **A** Distribution of post-translational modification sites of HASPIN analyzed via PhosphoSitePlus database, with x-axis being HASPIN residue number and y-axis total number of modification references (blue dots for phosphorylation, green for acetylation, red for ubiquitination). **B**, **C** Co-IP analysis showed the presence of lysine acetylation on HASPIN and H3 in HEK-293T and MDA-MB-231 cells, performing with anti-AcK (lysine acetylation). **D**, **E** Co-IP analysis showed the presence of lysine acetylation on HASPIN and H3 in HEK-293T and MDA-MB-231 cells after TSA/NAM treatment, performing with anti-AcK (lysine acetylation). **F** Co-IP analysis showed interactions of HASPIN with different acetyltransferases in HEK293T cells, transfecting with Myc-HASPIN and different acetyltransferase plasmids, performing with anti-Flag. **G**–**I** Endogenous interaction analysis of KAT5 and HASPIN in different BC cell lines (MDA-MB-231, BT549, MCF7) via Co-IP performing with anti-KAT5 or anti-HASPIN. **J**, **K** Exogenous interaction analysis of HASPIN and OTUB2 in HEK-293T and MCF7 cells, via Co-IP performing with anti-Myc, and transfecting with HA-OTUB2, Myc-HASPIN (wild-type, K751R, or K751Q). **L**, **M** Ubiquitination analysis in MCF7 and HEK-293T cells treated with MG132, transfected with Myc-HASPIN (wild-type, K751R, or K751Q), His-Ub, and HA-OTUB2, performing with anti-Myc. **N**, **O** Ubiquitination analysis in MCF7 and HEK-293T cells treated with MG132, transfected with Myc-HASPIN, His-Ub, HA-OTUB2, and HA-OTUB2, performing with anti-Myc.

Since the frequent occurrence of “crosstalk” between PTMs in physiological and pathological processes of cells, we wondered whether HASPIN was regulated by other PTMs. Proteomic database analysis revealed multiple acetylation sites in HASPIN, including a highly conserved acetylation site lysine 751(K751) (Fig. 6A). We preliminarily used anti-lysine acetylated antibody (AcK) in Co-IP to validate the presence of acetylation on HASPIN protein (Fig. 6B, C). The broad-spectrum HDAC deacetylases inhibitor TSA, and the SIRT deacetylases inhibitor NAM were applied for detecting whether HASPIN was regulated by acetylation in MDA-MB-231 and HEK-293T cells, and the subsequent IP assays showed that the acetylation level of endogenous HASPIN was significantly increased (Fig. 6D, E). We constructed and transfected Myc-tagged HASPIN wild-type (WT), HASPIN K751R [inactivation mutation to mimic deacetylation, lysine(K) to arginine(R)] and HASPIN K751Q [activation mutation to mimic acetylation, lysine(K) to glutamine (Q)] plasmids, and performed co-IP to confirm that mutations at K751 could affect the combination between HASPIN and OTUB2 (Fig. 6J, K and Supplementary Fig. S5C, D). The Ub-IP also revealed that mutations at K751 of HASPIN protein could change its’s ubiquitination level in MCF7 and HEK-293T cells (Fig. 6L, M). The above results proved that acetylation of HASPIN could improve its protein-binding ability with OTUB2, as well as the deubiquitylation level and stability of HASPIN protein.

Then, we performed a systematic IP experiment to screen out the upstream acetyltransferase of HASPIN, which demonstrated that, among five common candidate acetyltransferases (KAT2A, KAT2B, KAT5, CBP, and P300), only KAT5 covered the ability to bind with HASPIN in HEK-293T cells (Fig. 6F). The Co-IP assay verified the endogenous binding of KAT5 and HASPIN in BC cell lines (Fig. 6G–I). We also proved that KAT5 could enhance the acetylation level of HASPIN protein in MCF7 and HEK-293T cells (Supplementary Fig. S5A, B). Furthermore, we evaluated the function of acetyltransferases KAT5 on stabilizing HASPIN protein. We transfected cells with Myc-HASPIN, His-Ub, HA-OTUB2, and Flag-KAT5, and clarified that KAT5 could decrease the ubiquitination level of HASPIN protein (Fig. 6N, O). We performed protein degradation experiments under treatment with CHX, and expectedly discovered that KAT5 could strengthen the stability of HASPIN, whereas deacetylation of HASPIN showed instability (Supplementary Fig. S5E–H). The endogenous interaction between Myc-tagged HASPIN-WT, HASPIN-K751R, HASPIN-K751Q, and HA-OTUB2 was further validated by in-situ PLA in BC cells, which further confirmed the enhancement effects of acetylation on HASPIN-OTUB2 binding (Supplementary Fig. S5I). To clarify whether KAT5 enhanced acetylation of HASPIN through K751, we firstly perform Co-IP in HEK-293T cells transfected with Myc-HASPIN or Myc-HASPIN K751R, and Flag-KAT5, which revealed that KAT5 could not elevate the acetylation level of HASPIN protein in HASPIN K751R mutant subgroup (Supplementary Fig. S5J). We then treated HEK-293T cells with NU-9056 (a selective KAT5 histone acetyltransferase inhibitor) or DMSO (as blank control), and transfected with Myc-HASPIN, Flag-KAT5. The Co-IP results demonstrated that the KAT inhibitor could significantly restrain the acetylation level of HASPIN (Supplementary Fig. S5K). Taken together, the above results indicated that KAT5-mediated acetylation of HASPIN at K751 site possibly promoted OTUB2-HASPIN protein interaction, attenuated polyubiquitination level, and enhanced stabilization of HASPIN protein.

### OTUB2 and HASPIN protein levels are positively correlated and predict poor prognosis in BC patients

**Fig. 7:**
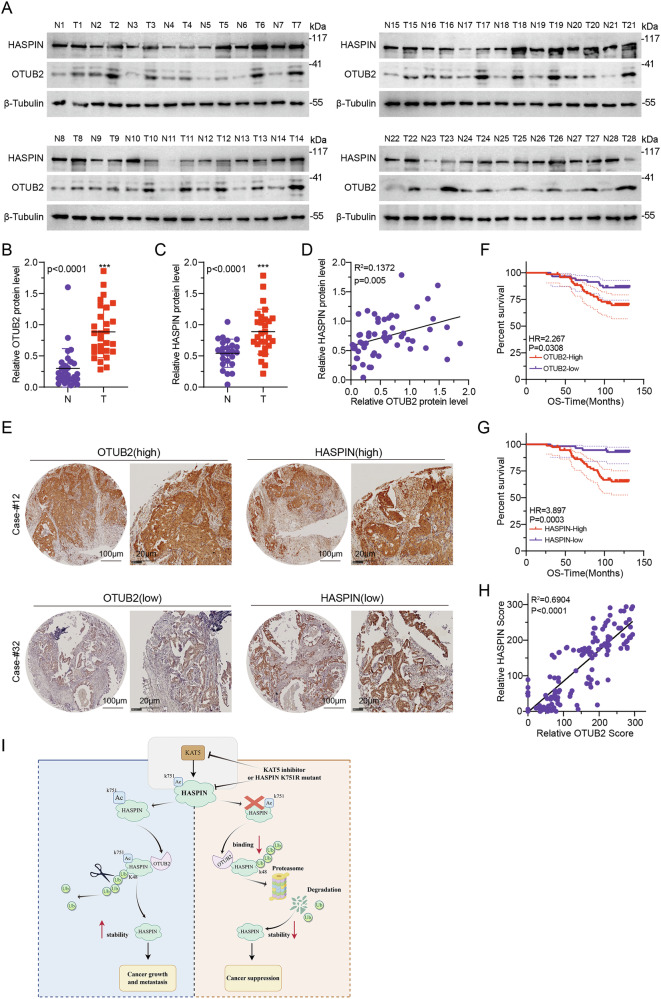
OTUB2 and HASPIN protein levels are positively correlated and predict poor prognosis in BC patients. **A** Western-blotting was used to detect the expressions of HASPIN, OTUB2, and β-Tubulin (loading control) proteins in normal (N1-N28) and tumor (T1-T28) tissues. **B**, **C** Box-plot of relative OTUB2 and HASPIN protein levels in normal (N) and tumor (T) tissues with each dot representing a sample. **D** Scatter plot of the correlation between relative HASPIN and OTUB2 protein levels. **E** Representative immunohistochemistry images of high and low expressions of OTUB2 and HASPIN proteins in tissue sections. Scale bar, 100 μm (left) and 20 μm (right). **F**, **G** Kaplan-Meier survival curves of overall survival (OS) for patients with high and low OTUB2 and HASPIN protein expressions. **H** Scatter plot of the correlation between relative HASPIN and OTUB2 scores. **I** Model showing that OTUD2 promotes HASPIN deubiquitination and promotes BC progression. ***p < 0.001.

Our research ultimately settles on the clinical relevance of specific proteins. Firstly, we analyzed the protein expression patterns of HASPIN and OTUB2 in BC patients, which showed that both proteins were upregulated in tumor tissues compared with para-cancerous normal tissues, pointing out their potential function in tumor promotion (Fig. 7A–C). We subsequently analyzed the correlation of OTUB2-HASPIN axis in BC patients, which showed positive relationship between the two protein levels, both by immunoblot analysis in clinical samples (Fig. 7D) and IHC staining assays in tissue microarrays (Fig. 7E, H). Kaplan–Meier survival analysis displayed that either OTUB2 or HASPIN protein high expression was positively related to poor overall survival (OS) in BC patients (Fig. 7F, G). Collectively, these results strongly validate OTUB2-dependent stabilization of HASPIN.

## Discussion

In this research, we identified HASPIN as a tumor promoter in BC development, especially in proliferation and metastasis of cancer cells, which was consistent with the previous study [25]. It is found that HASPIN possesses moderate conservation through evolution, and plays prominent role in eukaryotic organisms [26]. HASPIN is considered as a central modulator of cell proliferation and chromosome segregation, and is gradually proved to be implicated in cancers, not only its dysregulation in multiple cancer types, but also the relevance with patients’ survival [20–22, 27, 28]. Current evidence indicates HASPIN exerts dual tumor-promoting mechanisms. For instance, it modulates chromosome segregation by concurrently regulating chromosome passenger complex activity and cohesin stability, thereby preserving aberrant genetic material [26, 29–31]. Besides, HASPIN upregulation leads to increased H3-Thr3p levels and downregulation of ciliogenesis, which is likely related to cell cycle progression and carcinogenesis [32]. In addition, HASPIN-induced loss of cellular polarity could probably promote EMT, and thus contribute to tumor development [33, 34]. Though in early stage of tumor, loss of HASPIN contributes to genome instability and malignant transformation [26]. However, HASPIN overexpression favors tumor expansion in later stages, where the mechanism of increased HASPIN expression remains unknown.

With this above question, we further explored the upstream mechanism of HASPIN overexpression, which demonstrated that HASPIN protein level was partly regulated by deubiquitination modification, and the regulator of it was OTUB2. As a prominent Otubain subfamily member, OTUB2 deubiquitinates key proteins across diverse signaling pathways [35, 36], including the Hippo pathway [37], the nuclear factor kappa B (NF-kappaB) pathway [38, 39], the protein kinase B/mammalian target of rapamycin (Akt/mTOR) pathway [40], and the β-Catenin signaling [41] etc. In most cancer types, OTUB2 exerts tumor-promotional roles, such as promoting the proliferation, invasion and migration of cancer cells [42, 43], modulating the aerobic glycolysis [44], enhancing stemness feature and suppress chemosensitivity [45]. However, researchers also found that OTUB2 could suppress tumor development through inhibiting mitochondrial metabolic reprogramming [46], and increasing phosphatidylserine synthesis [47]. In addition, recent studies revealed that OTUB2 was a negative regulator of antitumor immunity, which directly interacted with PD-L1 and stabilized its expression in the endoplasmic reticulum. The inhibitor of OTUB2 successfully reduced PD-L1 expression in tumor cells and suppressed tumor growth [48]. These findings suggest that the complex effect of OTUB2 in cancer development mostly relays on its substrate proteins, which needs further research to elucidate its dual roles.

Through multiple verification, we identified HASPIN protein as the direct substrate of OTUB2, and both exerted positive roles in BC development. Overexpression of OTUB2 directly deubiquitinated HASPIN by removing the K48-linked polyubiquitin chain to protect HASPIN from proteasomal degradation, and led to its stabilization and accumulation in BC cells. Furthermore, we identified the protein kinase region and K618 residue of HASPIN were critical for their interactions. The tight relationship between HASPIN and OTUB2 protein levels was further clarified in clinical samples derived from BC patients.

Recent researches focused on the diversity and functionality of PTMs, especially the interaction between ubiquitination and other distinct PTMs, such as phosphorylation [49], acetylation [50], and SUMOylation [51] etc. The interactions between PTMs play vital roles in gene expression, genome structure, cell division and DNA damage response. Both ubiquitination and acetylation occur on lysine residue, and there exist multiple “crosstalk” between them under diverse biological background [9, 52]. Interestingly, we found that HASPIN had a highly conserved acetylation site K751. We proved the presence of acetylation on HASPIN protein, as well as its contribution to OTUB2-HASPIN combination. Furthermore, we found that lysine acetyltransferase KAT5 could induce HASPIN acetylation at K751 residue, and therefore enhanced OTUB2-induced HASPIN protein deubiquitination and stabilization. Functional analyses demonstrated that HASPIN acted as a tumor promoter to accelerate BC progression in a OTUB2-dependent manner both in vivo and in vitro. This KAT5- acetylation-OTUB2- deubiquitination-HASPIN oncogenic axis might exert critical role in BC.

Despite these appealing findings, we should acknowledge several limitations. Firstly, as a serine/threonine kinase, HASPIN itself could function as a regulator involved in mediating the stability of other proteins. A previous study reported the downstream pathway of HASPIN in BC progression. HASPIN was shown to promote BC pathogenesis via MDM2-dependent ubiquitination of E2F1 [25]. The targets or pathways being activated following HASPIN stabilization were not investigated in our study, and whether there exist other mechanisms of HASPIN-mediated tumor progression remain unclear. In addition, we only analyzed the effect of OTUB2-induced deubiquitination in protein expression and stabilization of HASPIN, the subcellular localization, structure alteration, and enzymatic activity of it are still unknown, which warrant further exploration. We also observed that, the persistence of HASPIN expression after OTUB2 knockdown likely reflects the fact that HASPIN protein homeostasis is governed by multiple, overlapping regulatory mechanisms. OTUB2-mediated deubiquitination is just one of these pathways. Other potential mechanisms that could sustain HASPIN levels, including transcriptional regulation, compensation by other DUBs, and differences in translation efficiency or inherent protein stability. Besides, variations in genetic background, signaling pathway activity, and redundant regulatory networks across different cell lines can significantly influence the strength of OTUB2-dependent HASPIN regulation. This highlights the context-dependent nature of HASPIN regulation and underscores the importance of systematically comparing different cellular models in future studies. Thirdly, our research had not involved existing inhibitors of HASPIN or OTUB2. The clinical application potential of HASPIN and OTUB2 warrants further exploration. Last, while our IHC data from a commercial tissue microarray underscores the clinical relevance of HASPIN, future studies utilizing larger, multi-center cohorts with detailed clinicopathological annotations are warranted to definitively establish its prognostic and predictive value across BC subtypes.

In summary, we found that HASPIN protein was upregulated in BC tissues. The DUB OTUB2 could bind with, deubiquitinated and stabilized HASPIN protein to promote BC progression. Additionally, KAT5-induced HASPIN acetylation strengthened the interaction between HASPIN and OTUB2 protein, resulting in the stabilization and accumulation of HASPIN in BC. The positive relationship between HASPIN and OTUB2 proteins expression, as well as survival relevance, were also observed in BC patients. Our results demonstrated that targeting HASPIN stabilization via OTUB2 inhibition represented a promising therapeutic strategy in BC.

### Ethics approval

The study involving human participants and animals was reviewed and approved by the ethics committee of The Affiliated Huaian No.1 People’s Hospital of Nanjing Medical University. Written informed consent was obtained from all patients. All methods were performed in accordance with the relevant guidelines and regulations.

## Supplementary information

Supplementary Figure Legends

Supplementary Figure S1

Supplementary Figure S2

Supplementary Figure S3

Supplementary Figure S4

Supplementary Figure S5

Supplementary Table S1

Supplementary Table S2

Supplementary Table S3

Original Western Blots

Quantification data of Western blots

## Data Availability

The data of LC-MS/MS are provided in the paper and its Supplementary Information.
