# Supplementary material for: KAT5-mediated acetylation enhances the deubiquitination of HASPIN by OTUB2 and promotes breast cancer progression
Source: Cell Death Dis. 2026 Mar 27;17(1):411. doi: 10.1038/s41419-026-08658-5 (PMC13144612; doi:10.1038/s41419-026-08658-5)
Supplement: Supplementary file 1 — Supplementary Figure Legends [file 41419_2026_8658_MOESM1_ESM.docx]

**SUPPLEMENTARY FIGURE LEGENDS**

**Fig. S1. OTUB2 interacts with HASPIN. (A)** Relative HASPIN mRNA levels varied in unpaired control tissues (Up CT, n=113) and breast cancer tissues (BCT, n=1113) from TCGA-BC database. **(B, C)** Representative images and quantification were presented for both the invasion and colony forming ability of MCF7 cells transfected with either a vector or a HASPIN overexpression plasmid. **(D)** Co-IP showed the interaction between HA-OTUB2 (wild-type or C51S mutant) and Myc-HASPIN in HEK-293T cells. **(E)** Presence of OTUB2 and HASPIN proteins in different BC cell lines and their intracellular localization were shown by Immunofluorescence (IF). **(F, G, H)** Line scan intensity plot of OTUB2 (green), HASPIN (red), and DAPI (blue) signals were shown in BC cells. Scale bar, 10 μm. **(I)** Interactions between OTUB2 and HASPIN in HEK-293T cells were detected by in situ proximity ligation assay (PLA). The nuclei were located by DAPI (blue), and the red color represents the binding sites of the two proteins. Scale bar, 10 μm. **(J)** Diagrammatic presentation of HASPIN protein full length and various deletion mutants. **(K)** Co-IP indicated interactions between different Myc-HASPIN mutants and HA-OTUB2 in HEK-293T cells. **(L)** Co-IP (with an alternative immunoprecipitation direction) of different Myc-HASPIN mutants and HA-OTUB2 in HEK-293T cells. ***p < 0.001.

**Fig. S2. OTUB2 mediates HASPIN protein expression. (A)** Results of potential specific lysine (K) residues on HASPIN that showed enriched ubiquitination from PhosphoSitePlus® PTM database. **(B)** Transfection of truncated plasmids of HASPIN domains to narrow down the range of potential ubiquitination sites. **(C)** Ubiquitination assays demonstrated that mutation of the specific sites (K618) significantly attenuated the polyubiquitination of HASPIN. **(D, E)** Western blot analyses were performed to detect the protein levels of OTUB2 and β-Tubulin (loading control) in MDA-MB-231 and BT549 cells transfected with Ctrl-Sh or OTUB2 shRNA plasmids (OTUB2 Sh_1, or OTUB2 Sh_2, OTUB2 Sh_3, OTUB2 Sh_4). **(F)** Western blot analyses were performed to detect the protein levels of OTUB2 and β-Tubulin in MCF7 cells transfected with HA-Vector, HA-OTUB2, or HA-OTUB2 C51S. **(G)** Western blot analyses were performed to detect the protein levels of HASPIN, OTUB2 and β-Tubulin in BT549 cells transfected with Ctrl-Sh, OTUB2 Sh_1, or OTUB2 Sh_2. **(H)** Western blot analyses were performed to detect the protein levels of Myc, OTUB2, HA, and β-Tubulin in MCF7 cells transfected with HA-Vector, Myc-HASPIN, and different dose of HA-OTUB2. **(I, J)** Western blot analyses were performed to detect the protein levels of HASPIN, OTUB2 and β-Tubulin in MDA-MB-231 and BT549 cells transfected with Ctrl-Sh, OTUB2 Sh_1, or OTUB2 Sh_2, and treated with Chloroquine (CQ, lysosome inhibitor).

**Fig. S3. OTUB2 targets HASPIN for K48-linked deubiquitination. (A)** Co-IP showed that OTUB2 could induce the deubiquitination of HASPIN protein in a dose-dependent manner in HEK-293T cells co-transfected with different doses of HA-OTUB2, Myc-HASPIN, His-Ubi and treated with MG132. **(B)** Co-IP revealed that OTUB2 could not induce the deubiquitination of HASPIN protein in HEK-293T cells co-transfected with Myc-HASPIN, His-Ubi K48R mutant, HA-OTUB2, and treated with MG132. **(C)** Co-IP revealed that OTUB2 could not induce the deubiquitination of HASPIN protein in MCF7 cells co-transfected with Myc-HASPIN, His-Ubi mutants (K6, K11, K27, K29, K33), HA-OTUB2, and treated with MG132.

**Fig. S4. OTUB2 promotes HASPIN-mediated migration in BC. (A, B)** Representative images and graphic representation of wound healing assays at 0 h and 48 h for different MCF7 cells transfected with vector, OTUB2, and HASPIN Sh_#1 plasmids. ***p < 0.001.

**Fig. S5. KAT5-mediated acetylation enhances the deubiquitination of HASPIN by OTUB2. (A, B)** Co-IP analysis showed KAT5 induced the acetylation on HASPIN protein in MCF7 and HEK-293T cells transfected with vector or Flag-KAT5, performing with anti-HASPIN. **(C, D)** Exogenous interaction analysis of HASPIN and OTUB2 in HEK-293T and MCF7 cells, via Co-IP performing with anti-HA, and transfecting with HA-OTUB2, Myc-HASPIN (wild-type, K751R, or K751Q). **(E, F)** Western blot showed the time-course of HASPIN and OTUB2 protein levels in MCF7 cells after CHX treatment, co-transfected with OTUB2, vector or Flag-KAT5 plasmids. **(G, H)** Western blot showed the time-course of Myc and HASPIN protein levels in MCF7 cells after CHX treatment, co-transfected with OTUB2, Myc-HASPIN WT or K751R plasmids. **(I)** Interactions between HA-OTUB2 and Myc-HASPIN (WT, K751R, K751Q) in HEK-293T cells were detected by in situ proximity ligation assay (PLA). The nuclei were located by DAPI (blue), and the red color represents the binding sites of the two proteins. Scale bar, 10 μm. **(J)** Co-IP analysis showed acetylation level of HASPIN in HEK-293T cells transfected with Myc-HASPIN of Myc-HASPIN K751R, and Flag-KAT5. **(K)** Co-IP analysis showed acetylation level of HASPIN in HEK-293T cells transfected with Myc-HASPIN, Flag-KAT5, and treated with DMSO or NU-9056. ***p < 0.001.
