# Supplementary figures and images for: KAT5-mediated acetylation enhances the deubiquitination of HASPIN by OTUB2 and promotes breast cancer progression
Source: Cell Death Dis. 2026 Mar 27;17(1):411. doi: 10.1038/s41419-026-08658-5 (PMC13144612; doi:10.1038/s41419-026-08658-5)

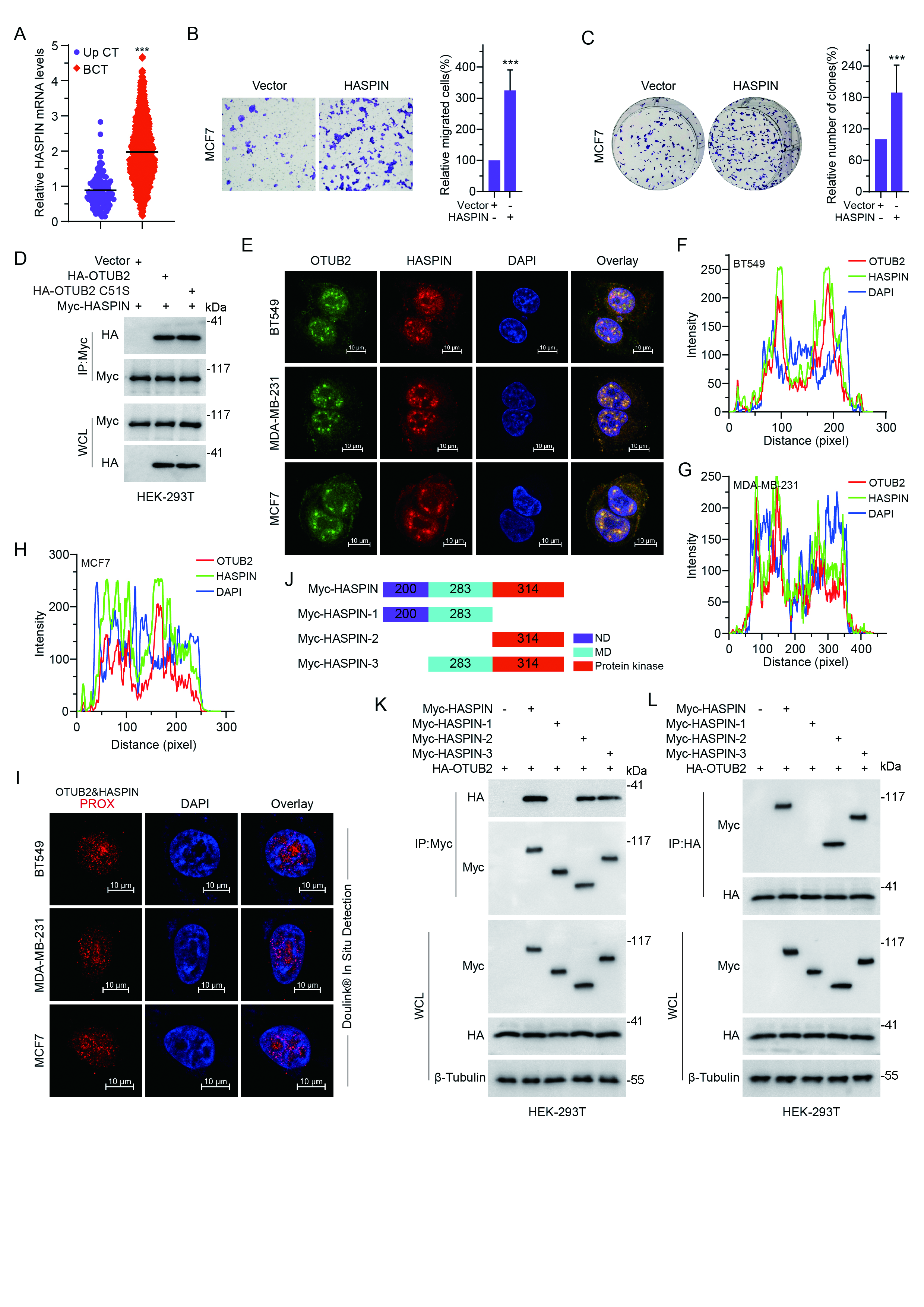

Supplement: Supplementary file 2 — Supplementary Figure S1 [file 41419_2026_8658_MOESM2_ESM.tif]

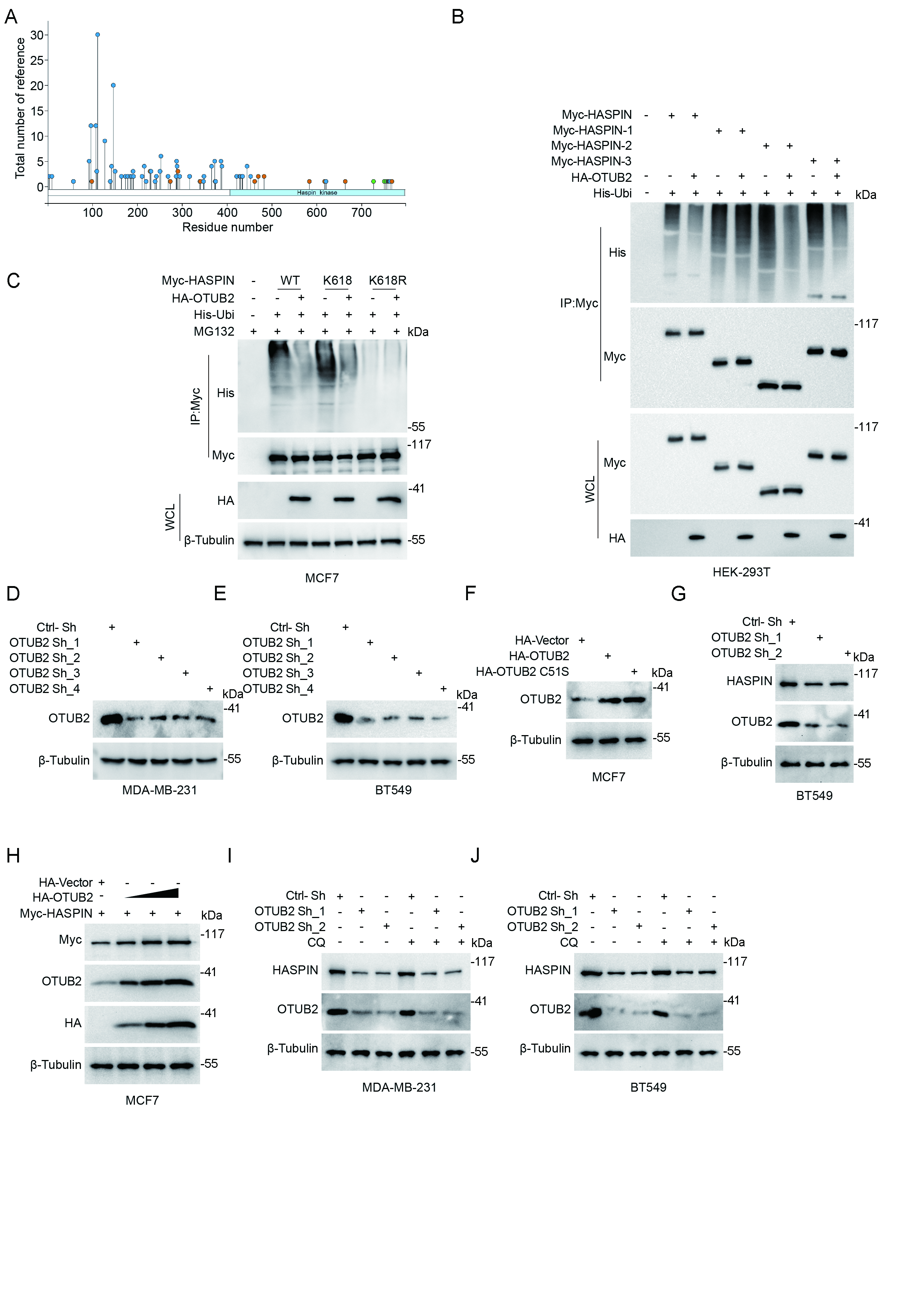

Supplement: Supplementary file 3 — Supplementary Figure S2 [file 41419_2026_8658_MOESM3_ESM.tif]

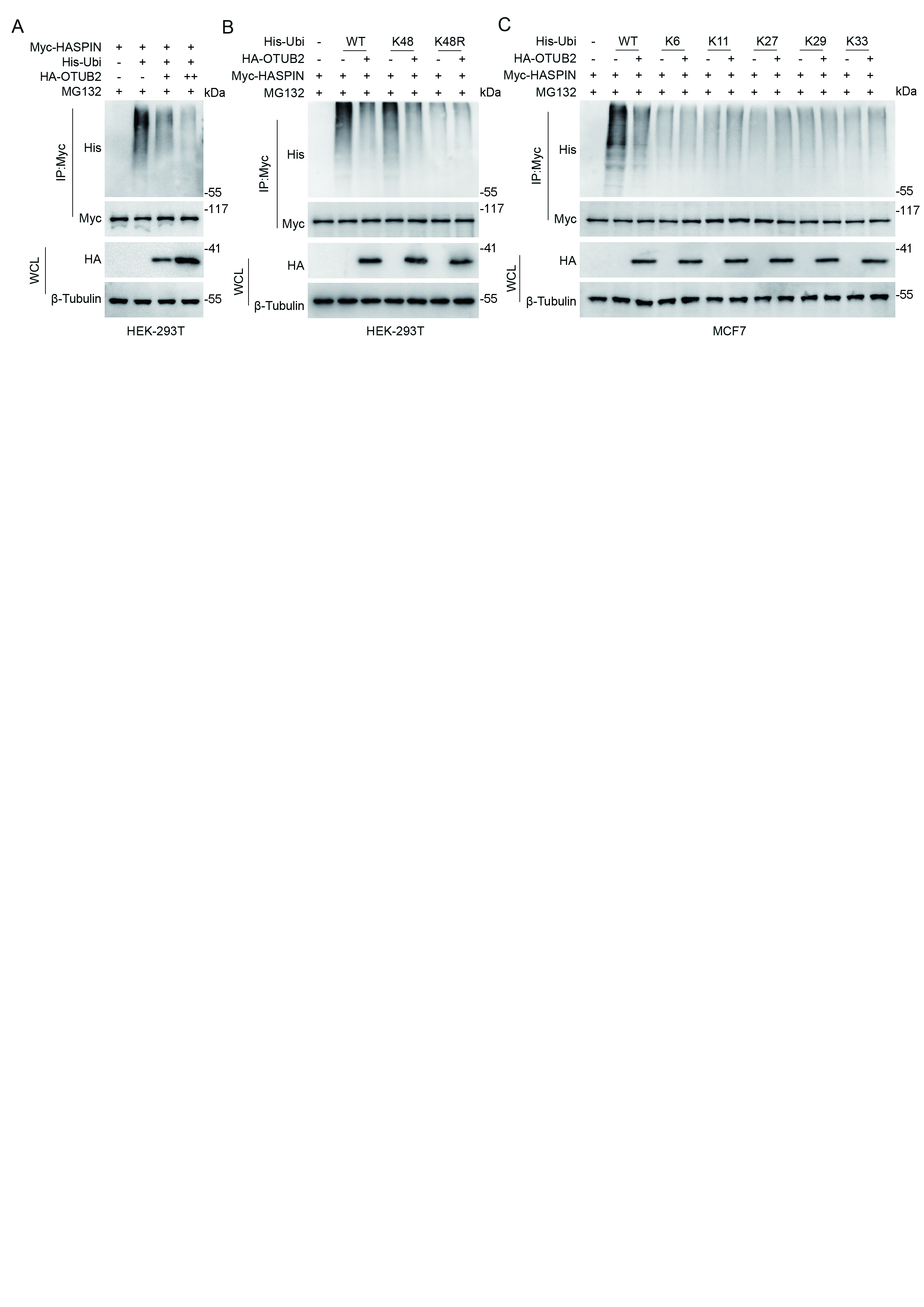

Supplement: Supplementary file 4 — Supplementary Figure S3 [file 41419_2026_8658_MOESM4_ESM.tif]

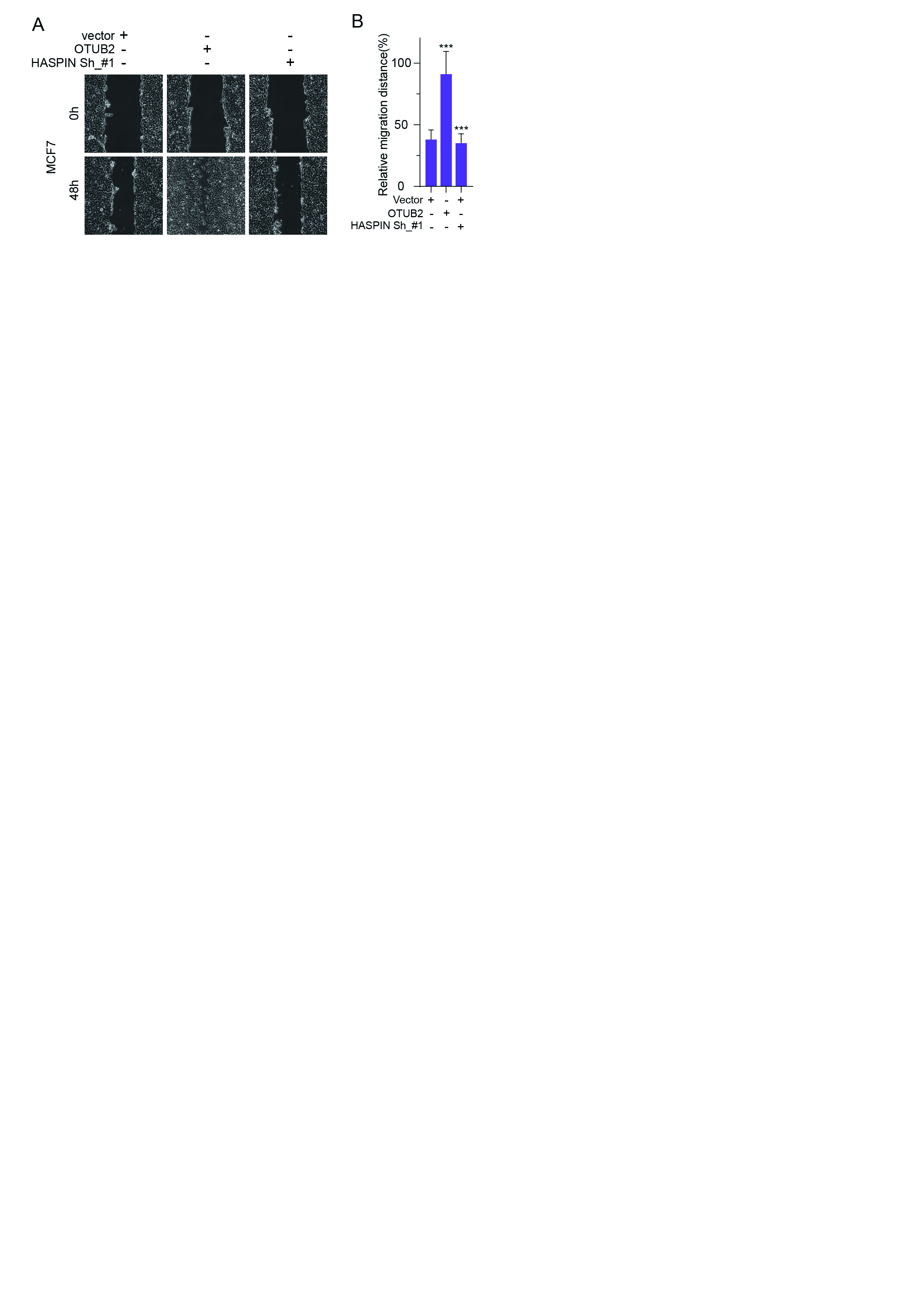

Supplement: Supplementary file 5 — Supplementary Figure S4 [file 41419_2026_8658_MOESM5_ESM.tif]

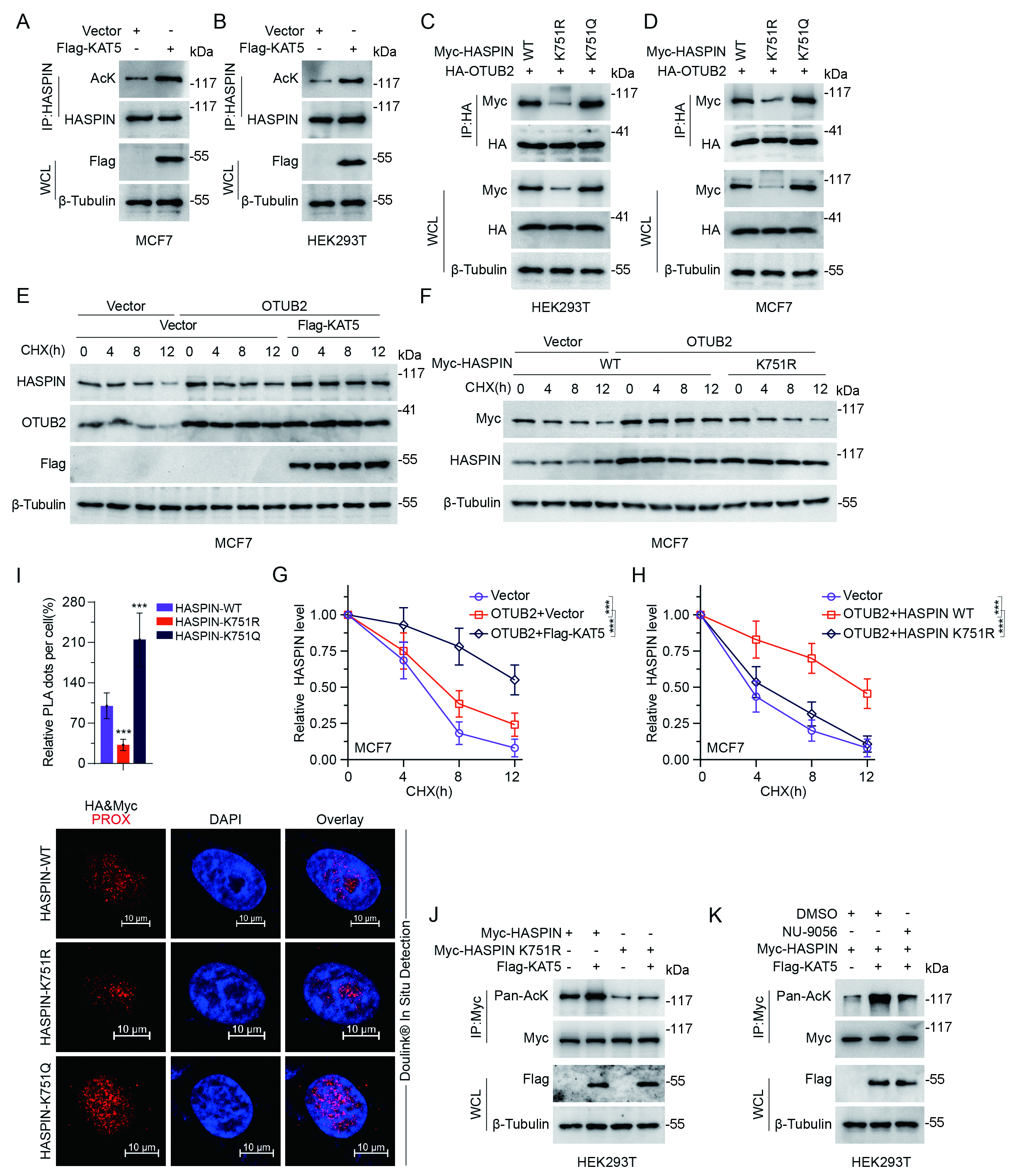

Supplement: Supplementary file 6 — Supplementary Figure S5 [file 41419_2026_8658_MOESM6_ESM.tif]
