# Supplementary material for: KAT5-mediated acetylation enhances the deubiquitination of HASPIN by OTUB2 and promotes breast cancer progression
Source: Cell Death Dis. 2026 Mar 27;17(1):411. doi: 10.1038/s41419-026-08658-5 (PMC13144612; doi:10.1038/s41419-026-08658-5)
Supplement: Supplementary file 7 — Supplementary Table S1 [file 41419_2026_8658_MOESM7_ESM.docx]

Supplementary Table S1. shRNA oligos sequences.

| Name | Sequence (5’-3’) |
| --- | --- |
| Sh-ctrl | AAACGCTCTCATCGACAAG |
| HASPIN Sh_#1 | AGGACTCTTGTCAAGAGAG |
| HASPIN Sh_#2 | AGGCTCATGAAGAAGGAGA |
| HASPIN Sh_#3 | ACAAATGACCTTCAAGACT |
| OTUB2 Sh_#1 | CCGTTTACCTGCTCTATAA |
| OTUB2 Sh_#2 | CAACCTCTCTTCCTCTATA |
| OTUB2 Sh_#3 | CCTTTAGTAGGAGCTCAAA |
| OTUB2 Sh_#4 | CCGATAAACATTGATTAAT |
