# Supplementary material for: KAT5-mediated acetylation enhances the deubiquitination of HASPIN by OTUB2 and promotes breast cancer progression
Source: Cell Death Dis. 2026 Mar 27;17(1):411. doi: 10.1038/s41419-026-08658-5 (PMC13144612; doi:10.1038/s41419-026-08658-5)
Supplement: Supplementary file 8 — Supplementary Table S2 [file 41419_2026_8658_MOESM8_ESM.docx]

Supplementary Table S2. Antibodies and reagents.

| **Antibodies** | | | |
| --- | --- | --- | --- |
| NAME | IDENTIFIER | RESOURCE | APPLICATION |
| HASPIN | A302-241A | Thermo Fisher | IHC, IP, WB |
|  | HPA030698 | Sigma-Aldrich | IF |
| OTUB2 | CF501942 | Origene | IF, WB |
|  | HPA002329 | Sigma-Aldrich | IHC |
|  | WH0078990M14 | Sigma-Aldrich | IP |
| KAT5 | sc-166323 | Santa Cruz Biotechnology | WB, IP |
| β-Tubulin | 10094-1-AP | Proteintech | WB |
| Ub | sc-8017 | Santa Cruz Biotechnology | WB |
| H3 | #9715 | Cell Signaling Technology | WB |
| AcK | sc-28336 | Santa Cruz Biotechnology | WB, IP |
| HA | #3724 | Cell Signaling Technology | WB, IP |
| Myc | #2276 | Cell Signaling Technology | WB, IP |
| His | #12698 | Cell Signaling Technology | WB, IP |
| Flag | #14793 | Cell Signaling Technology | WB, IP |

| **Chemicals** | | |
| --- | --- | --- |
| NAME | IDENTIFIER | RESOURCE |
| MG132 | HY-13259 | MCE |
| CHX | HY-12320 | MCE |
| CQ | HY-17589A | MCE |
| TSA | HY-15144 | MCE |
| NAM | HY-13515 | MCE |
| NU9056 | HY-110127 | MCE |
