# Supplementary material for: KAT5-mediated acetylation enhances the deubiquitination of HASPIN by OTUB2 and promotes breast cancer progression
Source: Cell Death Dis. 2026 Mar 27;17(1):411. doi: 10.1038/s41419-026-08658-5 (PMC13144612; doi:10.1038/s41419-026-08658-5)

1H

HASPIN

OTUB2

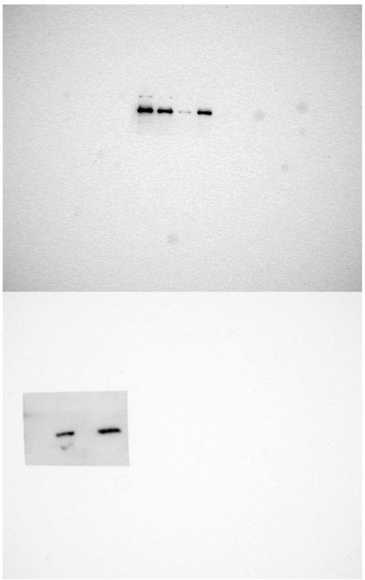

1I

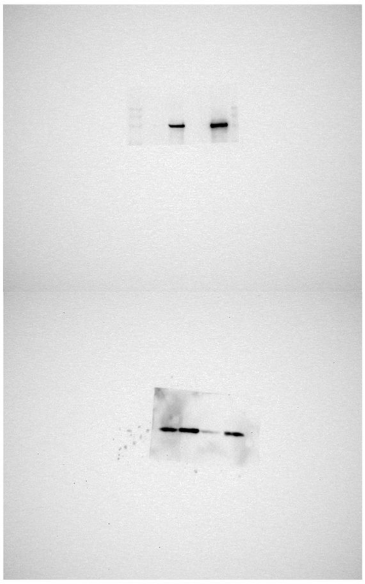

1J

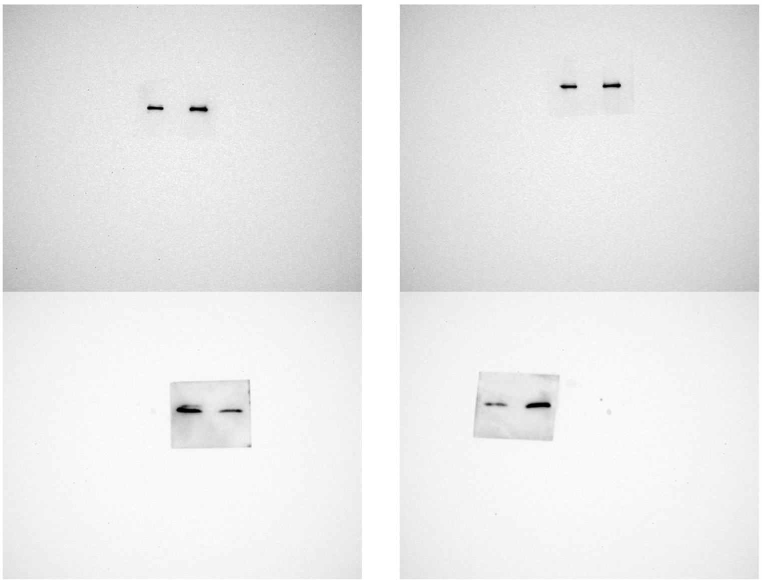

1K

HASPIN

OTUB2

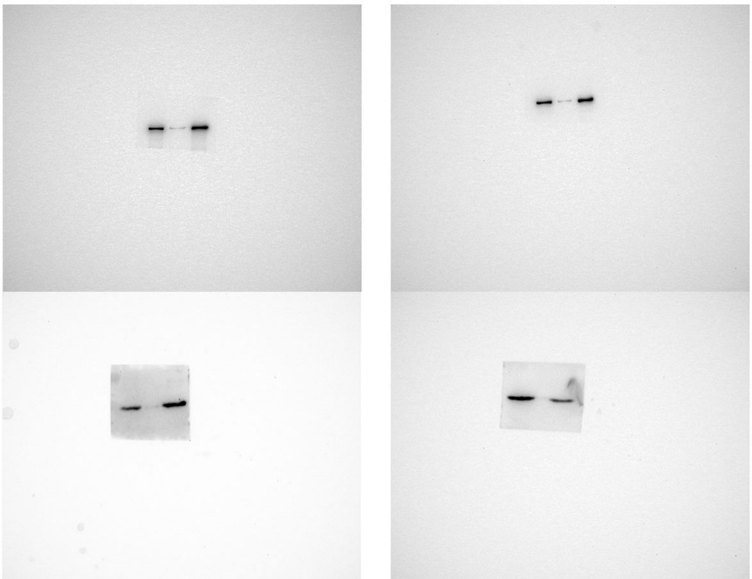

1L

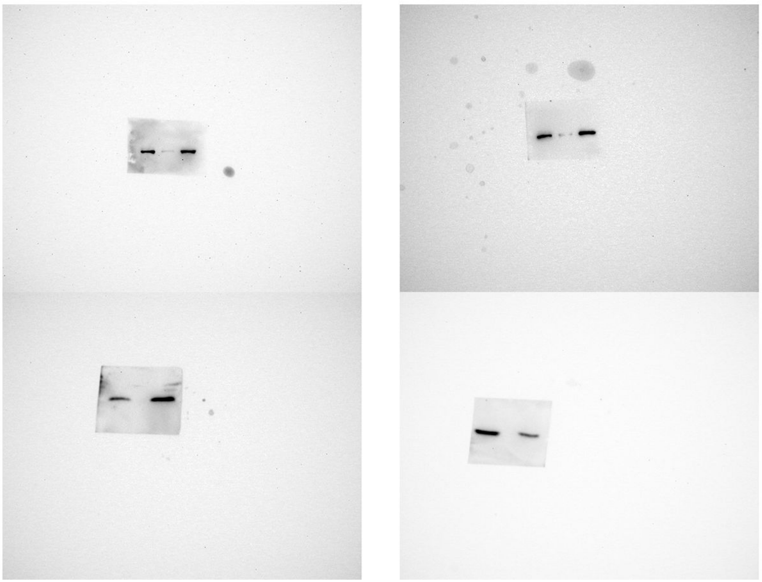

1M

HASPIN

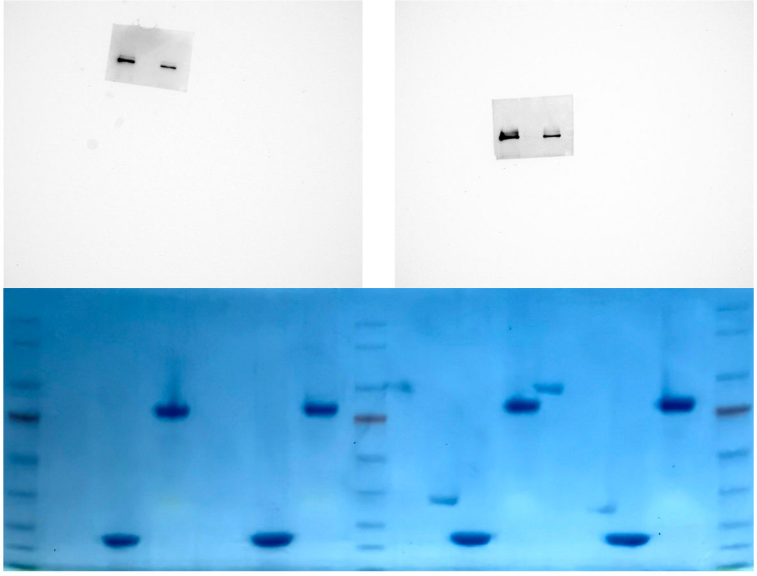

1N

OTUB2

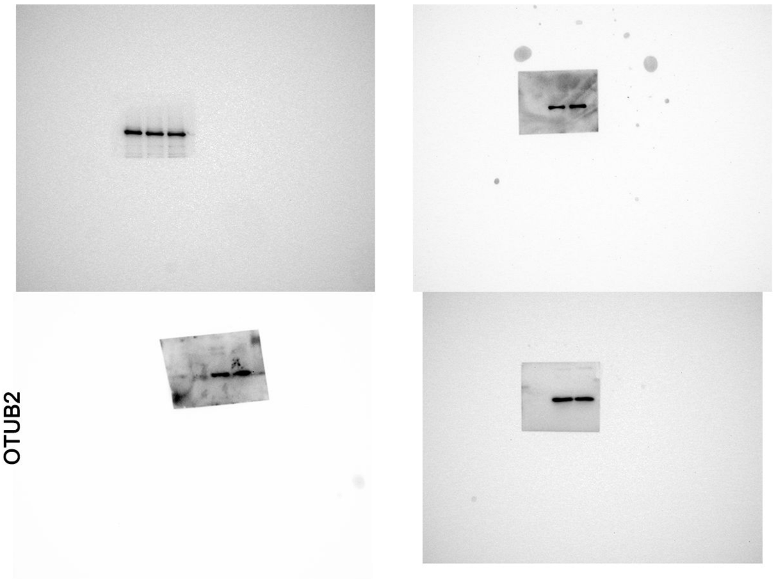

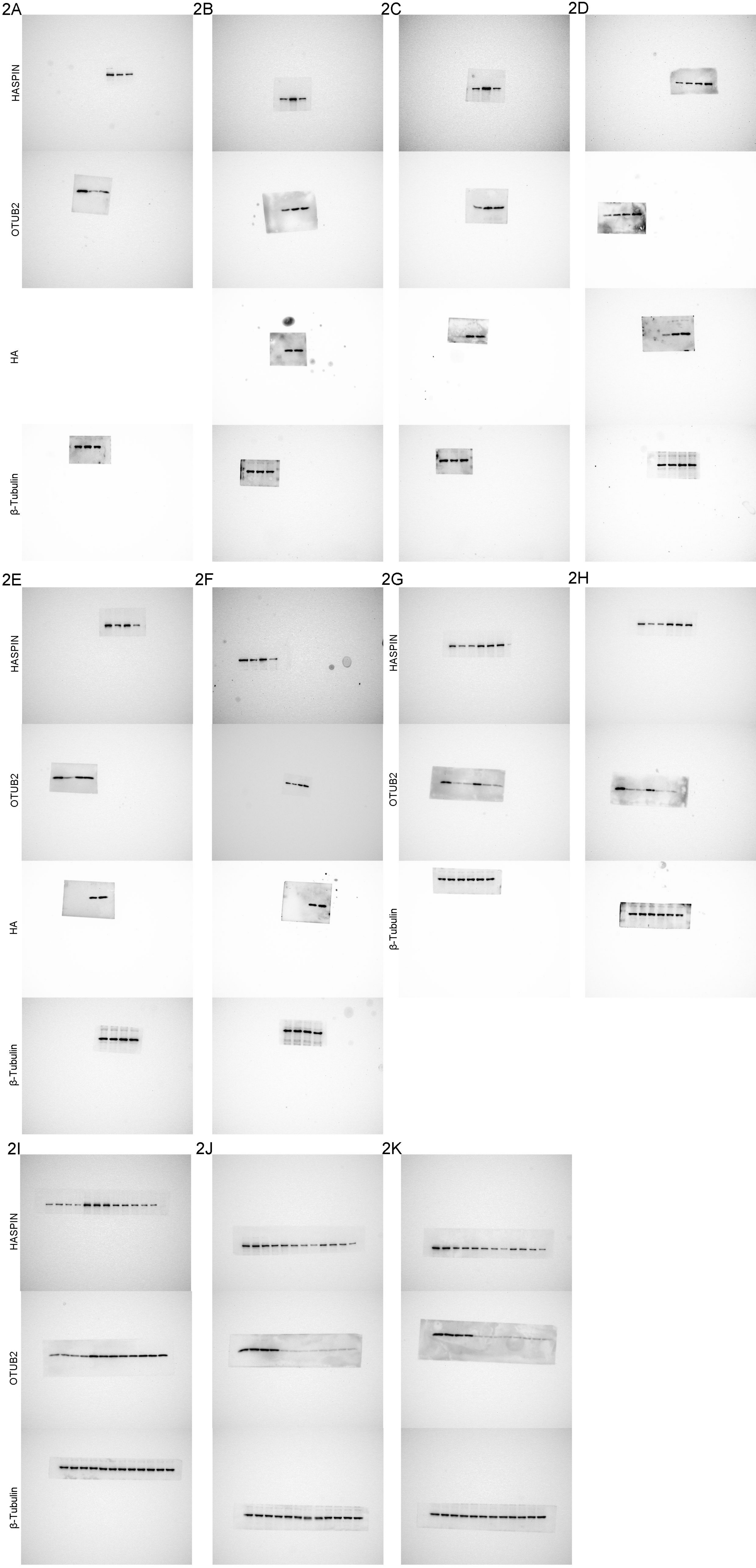
2A
2B
2C
2D
HASPIN
OTUB2
HA
β-Tubulin
2E
2F
2G
2H
HASPIN
OTUB2
HA
β-Tubulin
HASPIN
OTUB2
β-Tubulin
2I
2J
2K
HASPIN
OTUB2
β-Tubulin

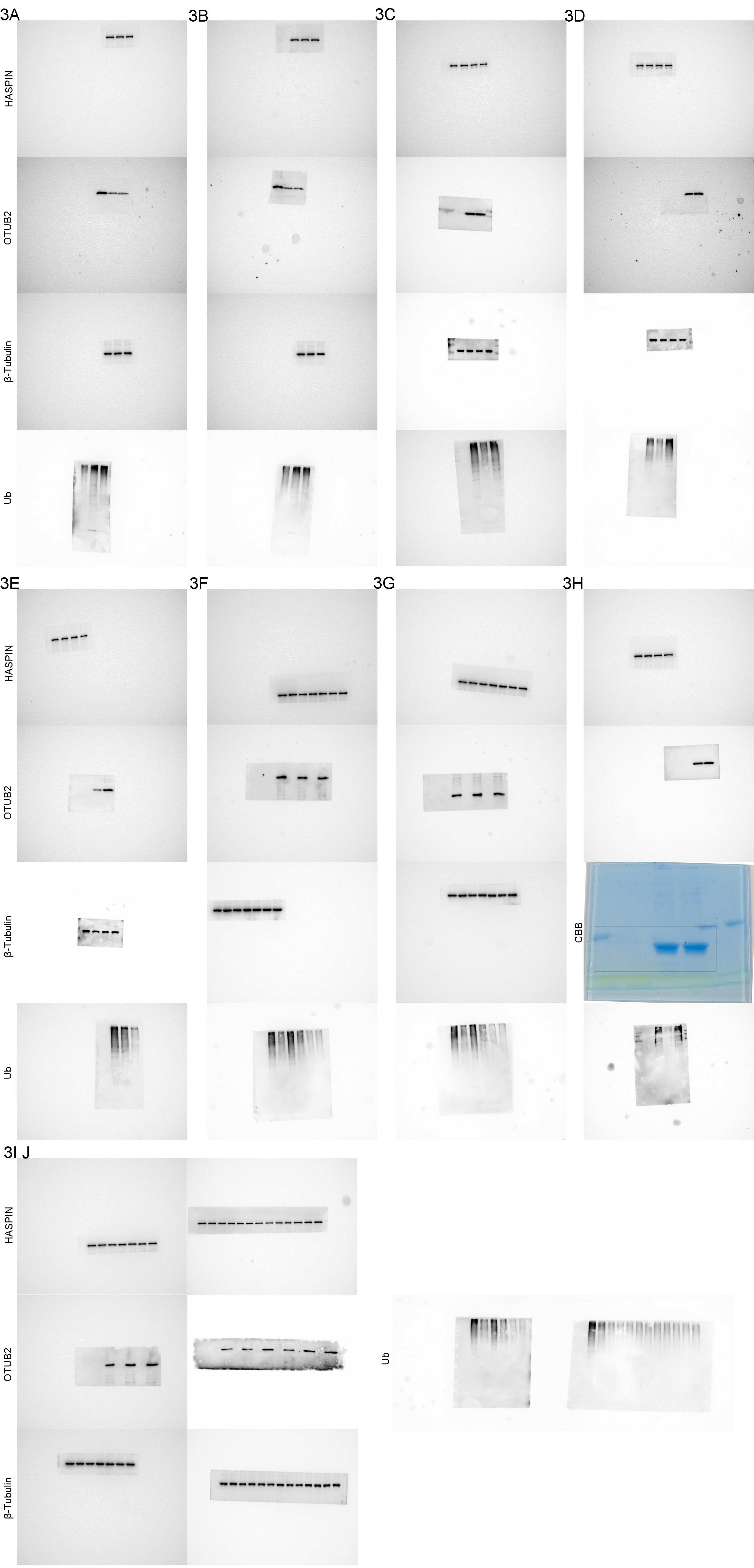
3A
3B
3C
3D
HASPIN
OTUB2
β-Tubulin
Ub
3E
3F
3G
3H
HASPIN
OTUB2
β-Tubulin
CBB
Ub
3I J
HASPIN
OTUB2
β-Tubulin
Ub

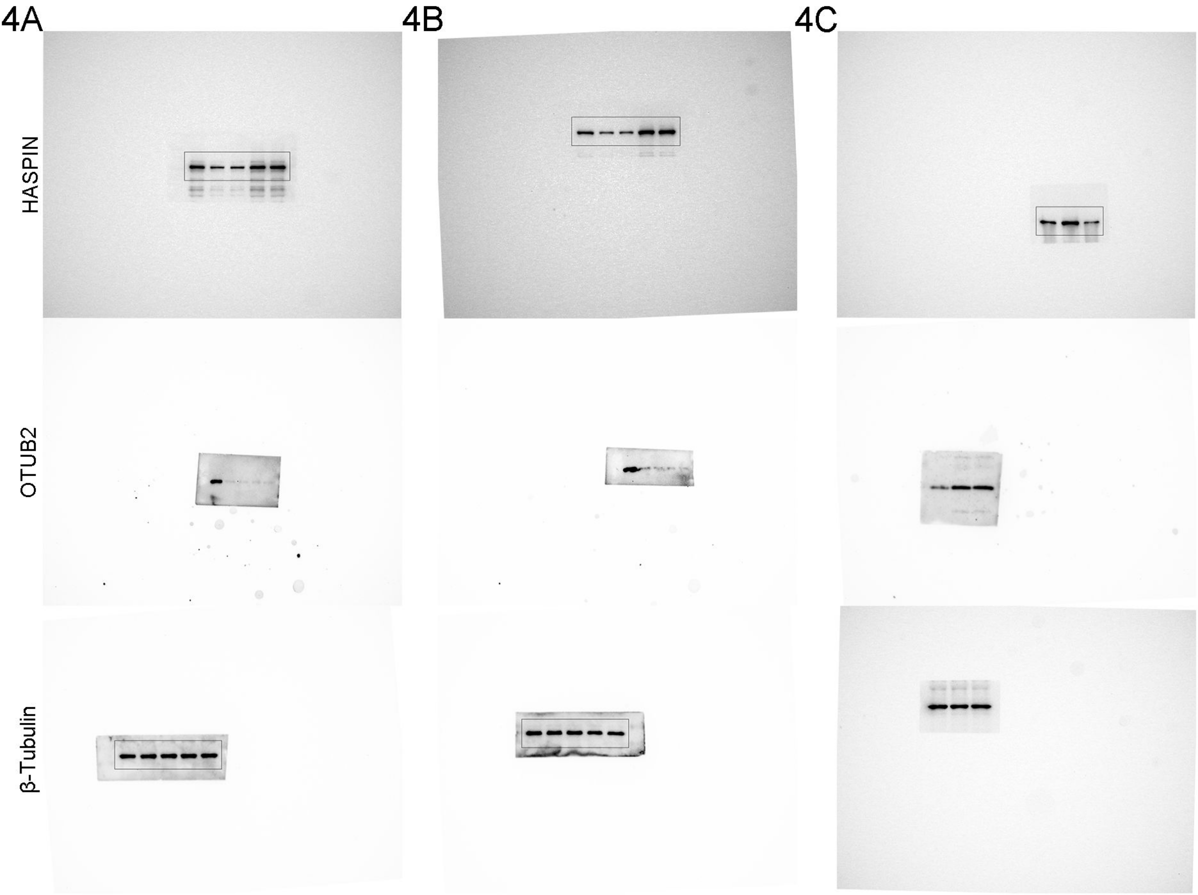
4A
4B
4C
HASPIN
OTUB2
β-Tubulin

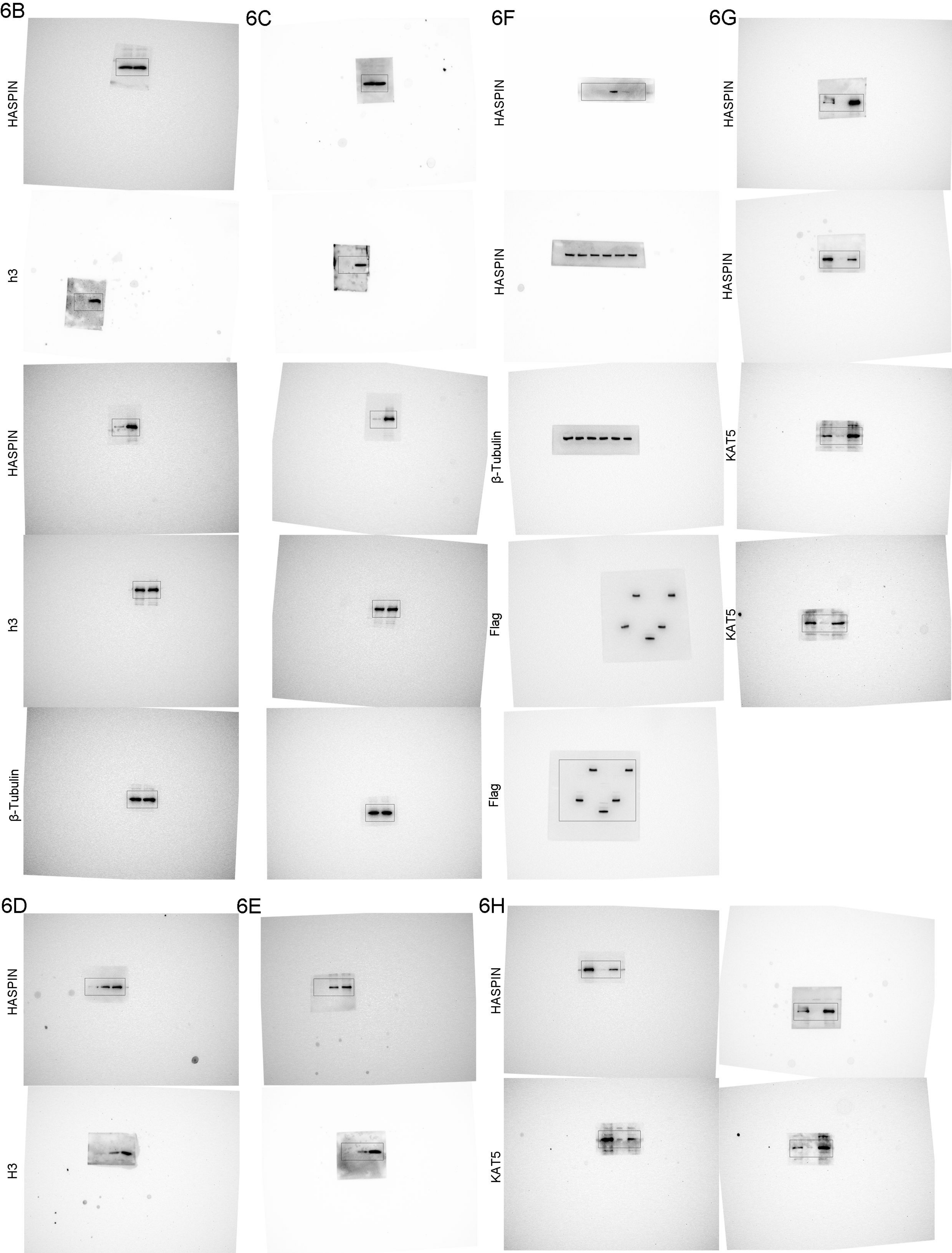
6B
HASPIN
h3
HASPIN
h3
β-Tubulin
6C
6F
HASPIN
HASPIN
β-Tubulin
Flag
Flag
6G
HASPIN
HASPIN
KAT5
KAT5
6D
HASPIN
H3
6E
6H
HASPIN
KAT5

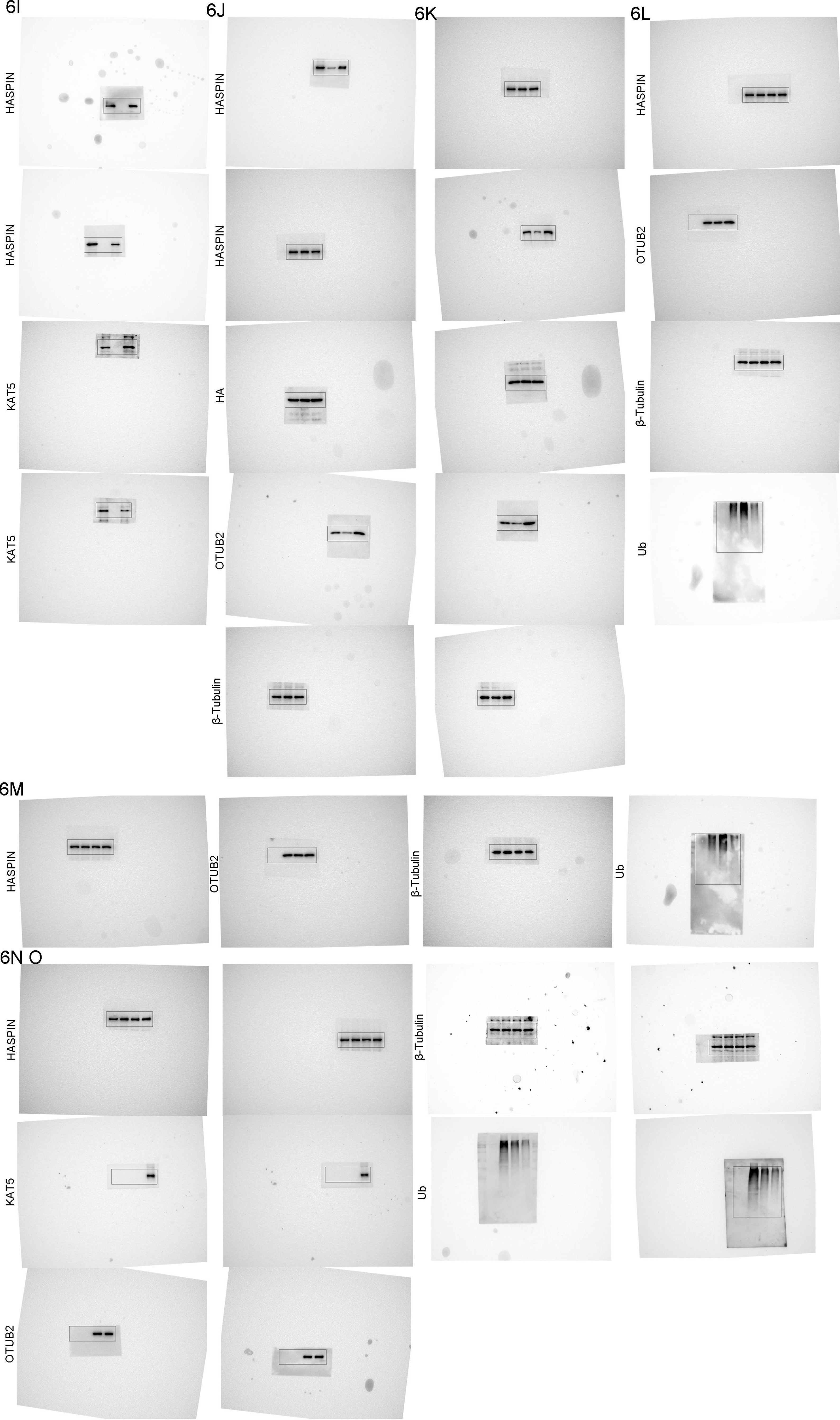
6I
HASPIN
HASPIN
KAT5
KAT5
6J
HASPIN
HASPIN
HA
OTUB2
β-Tubulin
6K
6L
HASPIN
OTUB2
β-Tubulin
Ub
6M
HASPIN
OTUB2
β-Tubulin
Ub
6N O
HASPIN
KAT5
OTUB2
β-Tubulin
Ub

7A

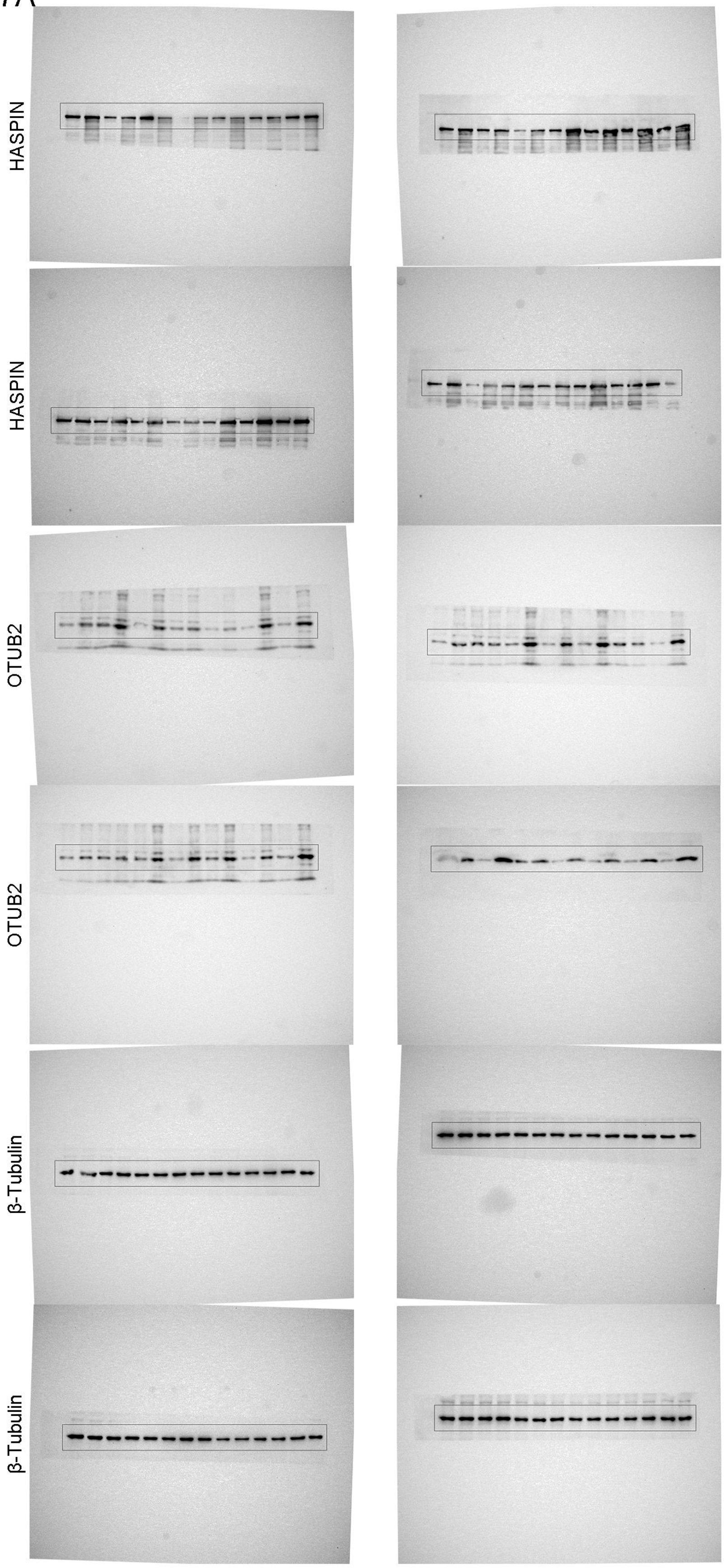

S1D

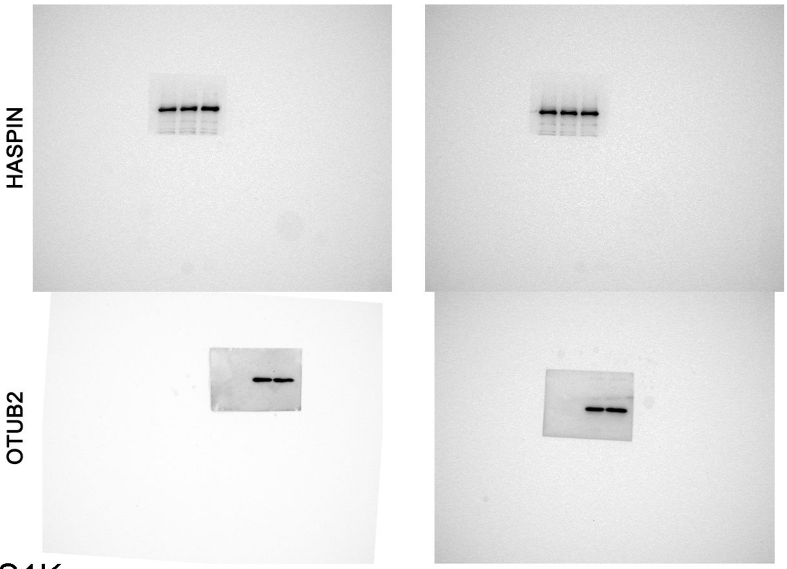
HASPIN
OTUB2

S1K

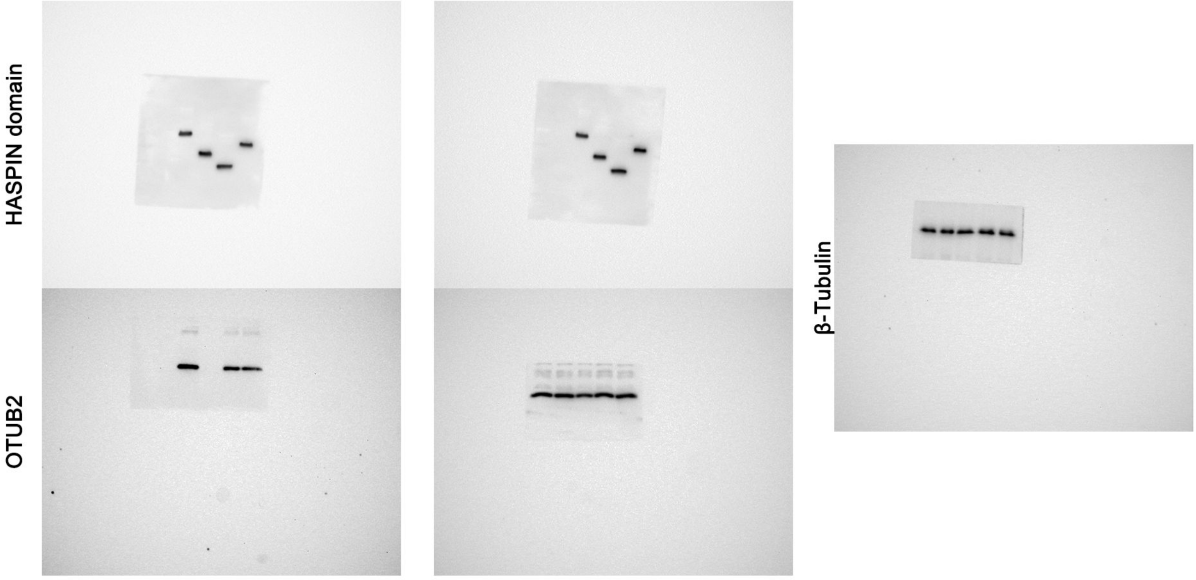
HASPIN domain
OTUB2
β-Tubulin

S1L

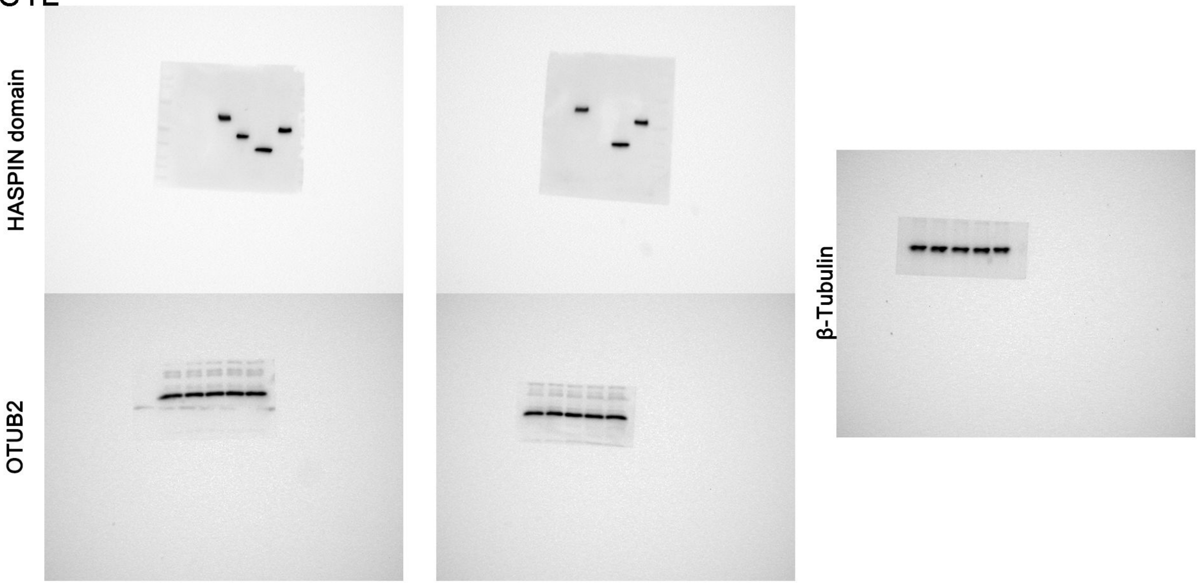
HASPIN domain
OTUB2
β-Tubulin

S2B

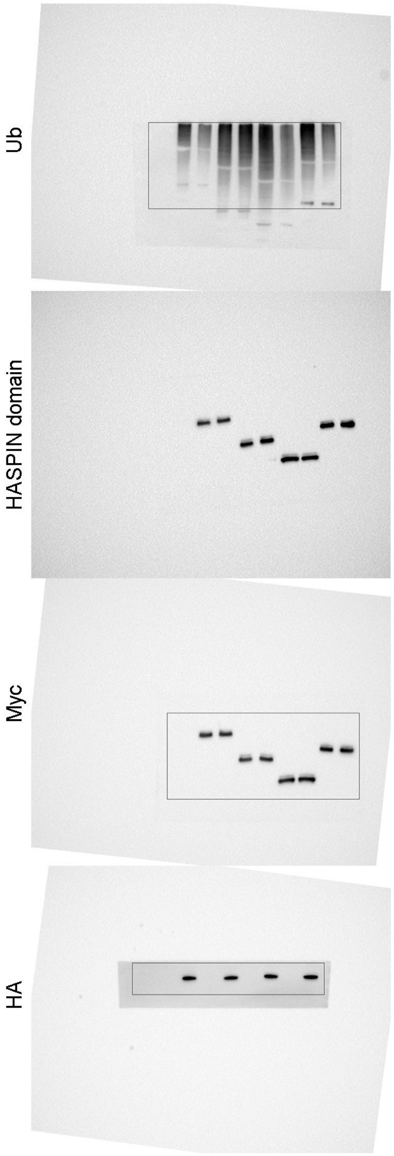

S2C

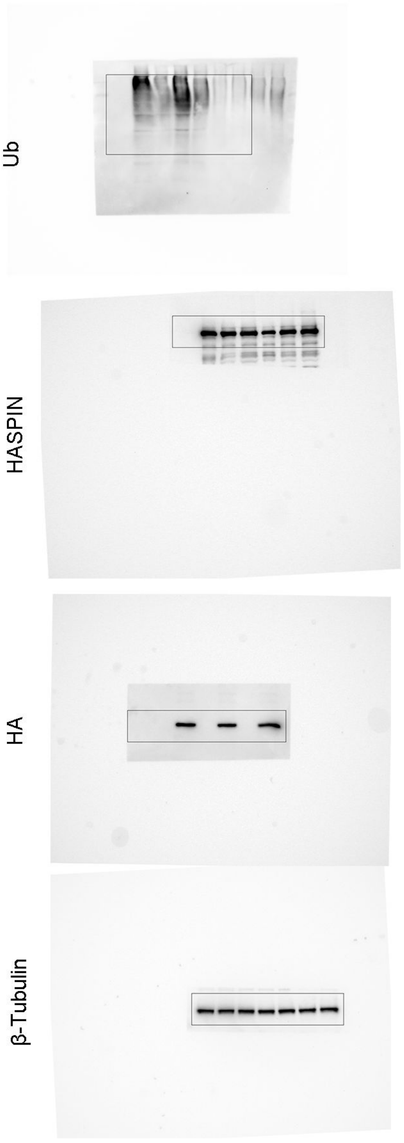

S2D

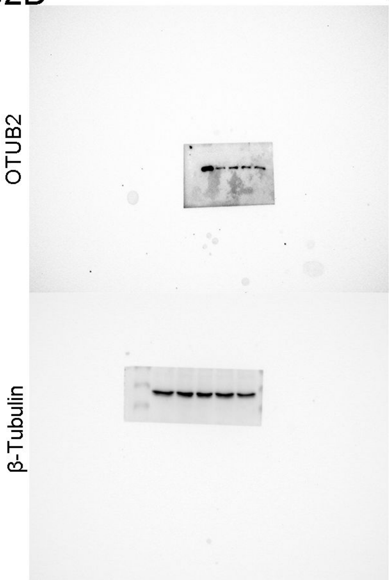

S2E

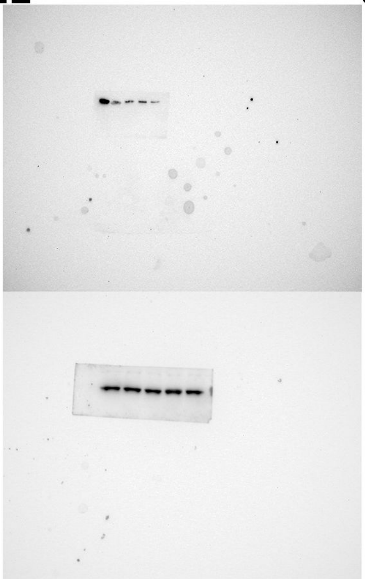

S2F

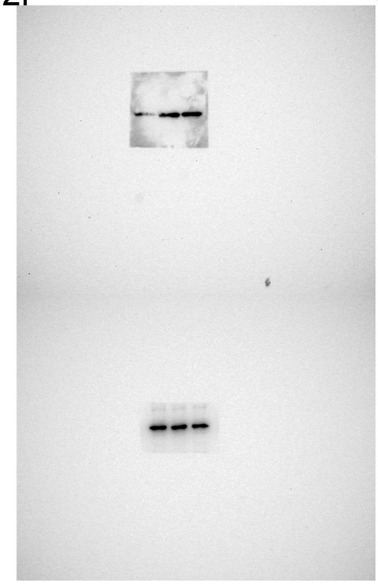

S2G

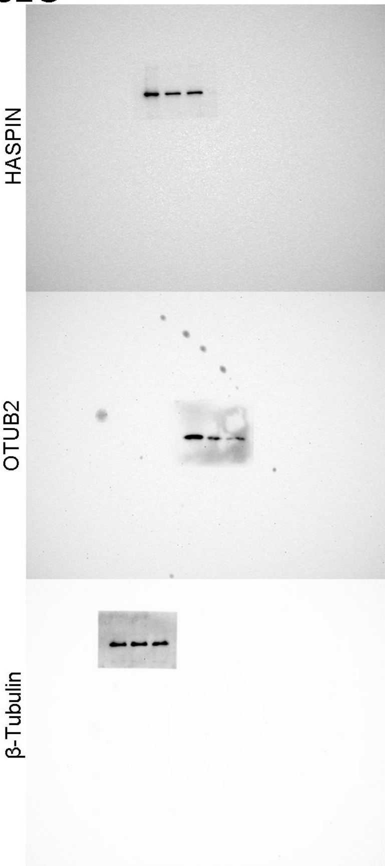

S2H

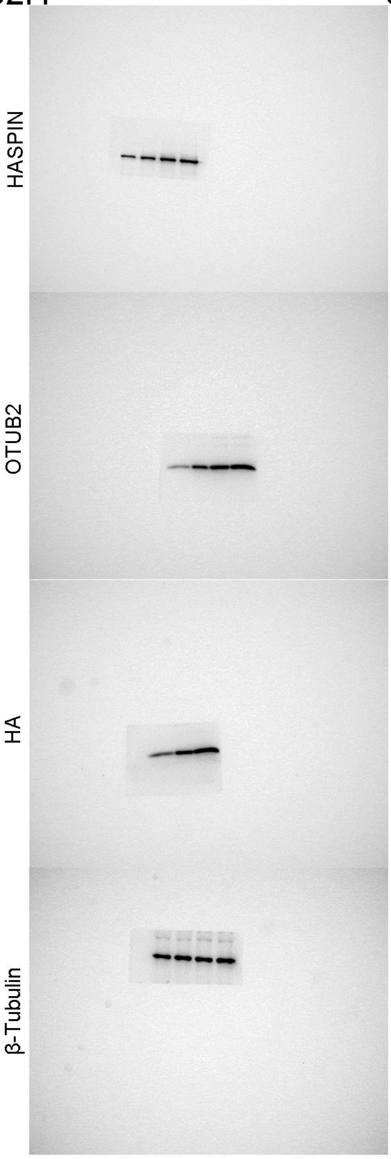

S2I

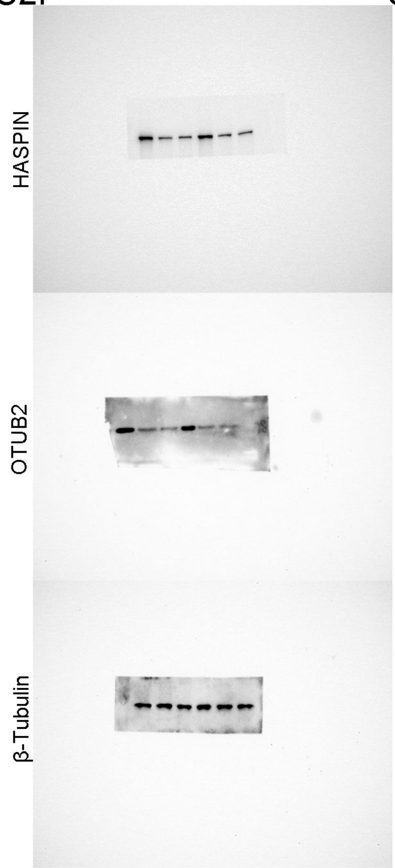

S2J

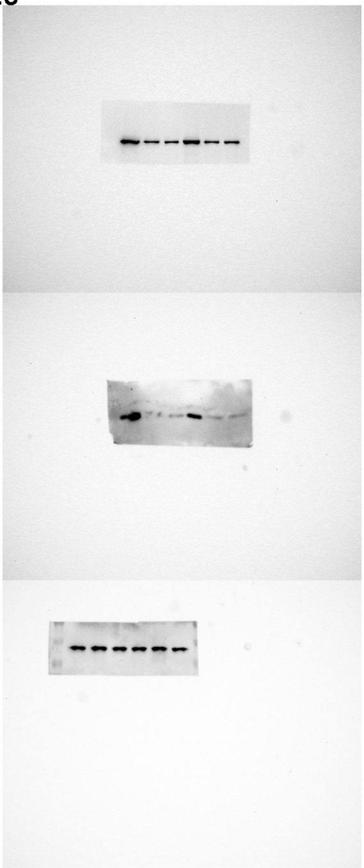

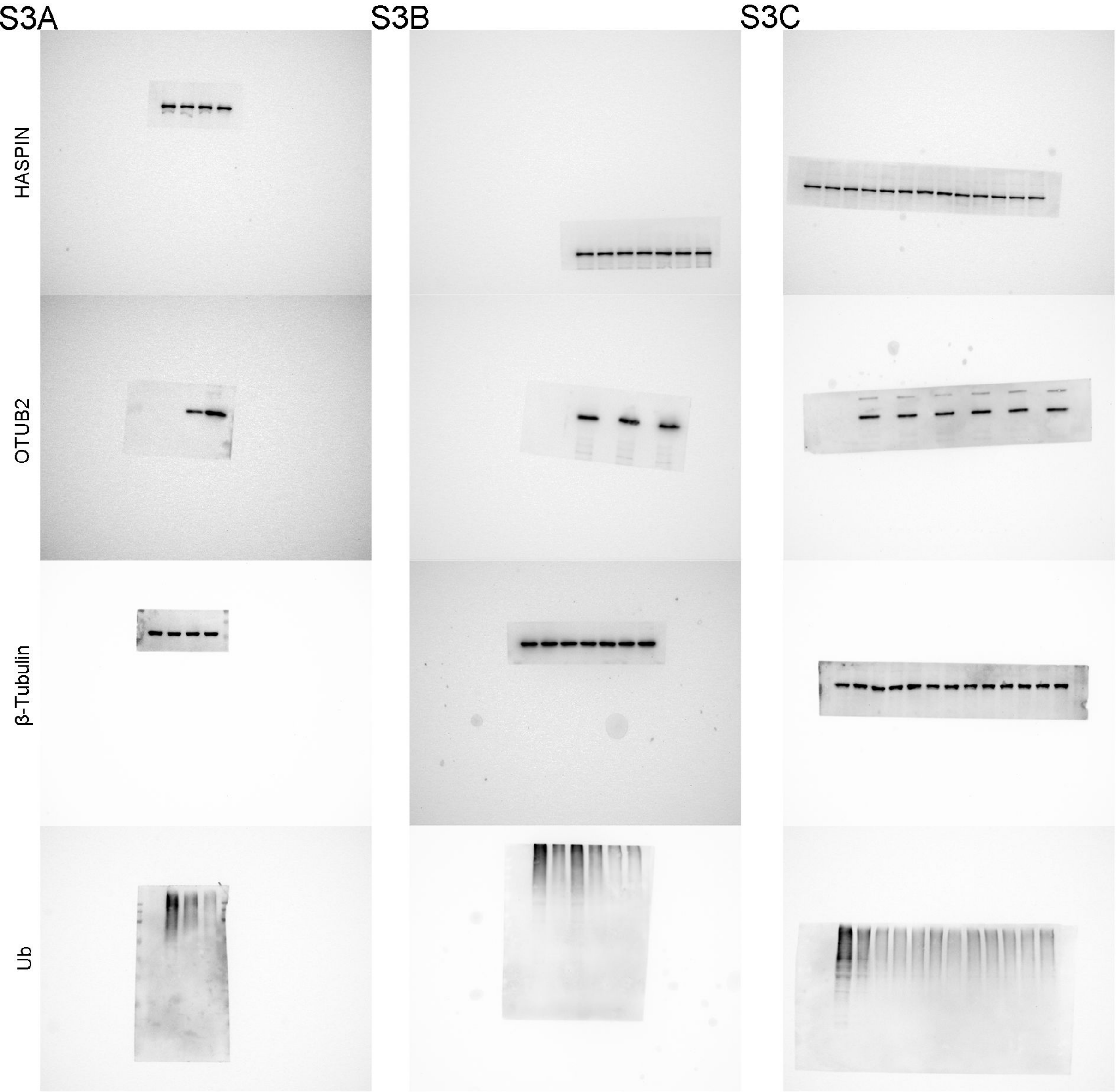
S3A
S3B
S3C
HASPIN
OTUB2
β-Tubulin
Ub

S5A

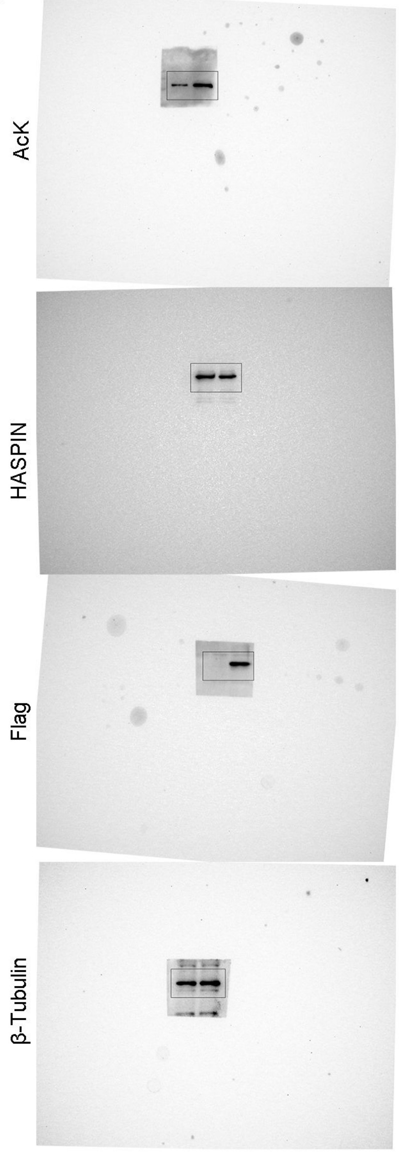

S5B

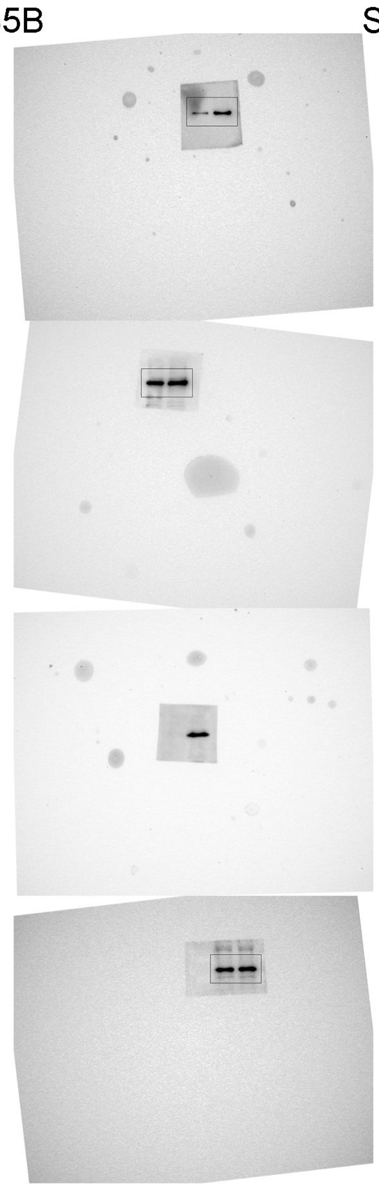

S5C

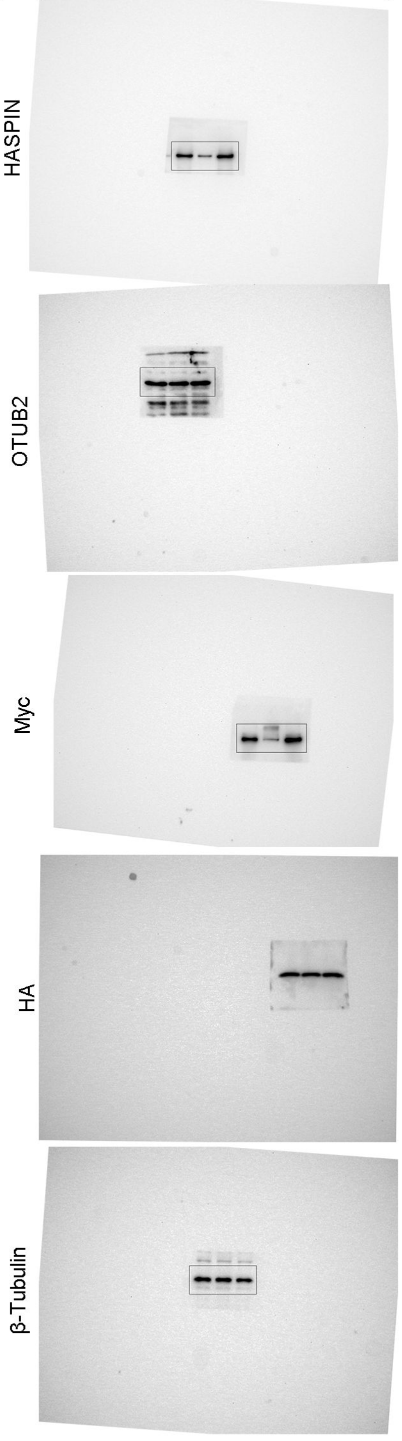

S5D

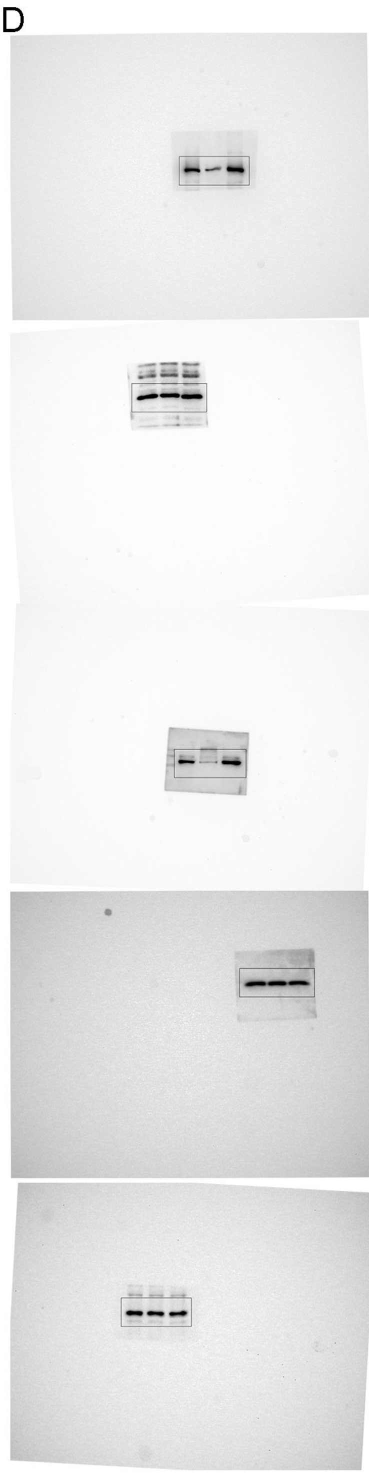

S5E

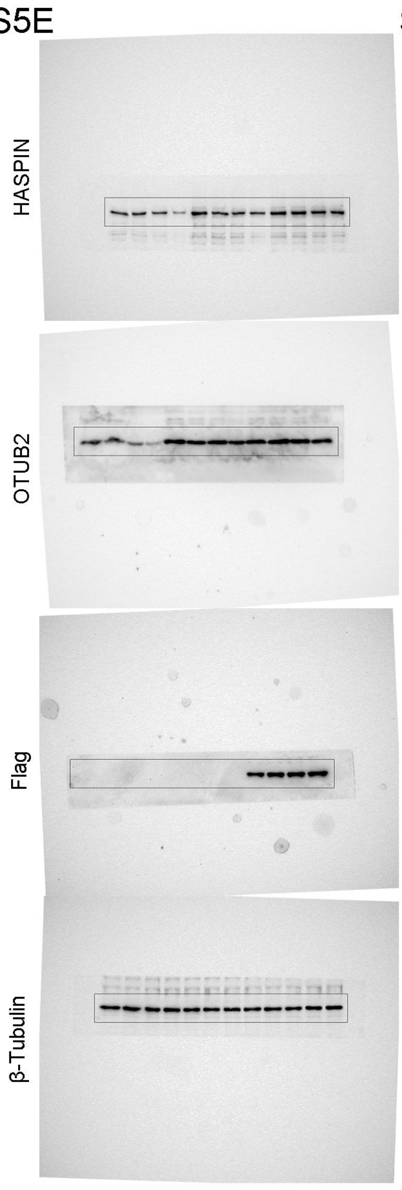

S5F

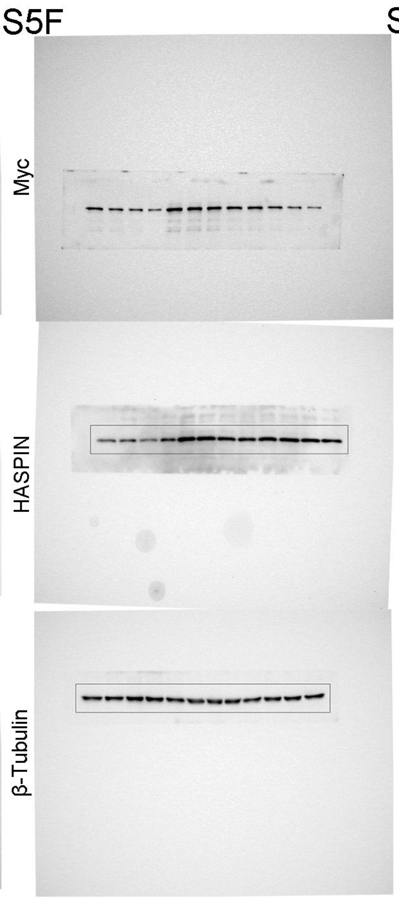

S5J

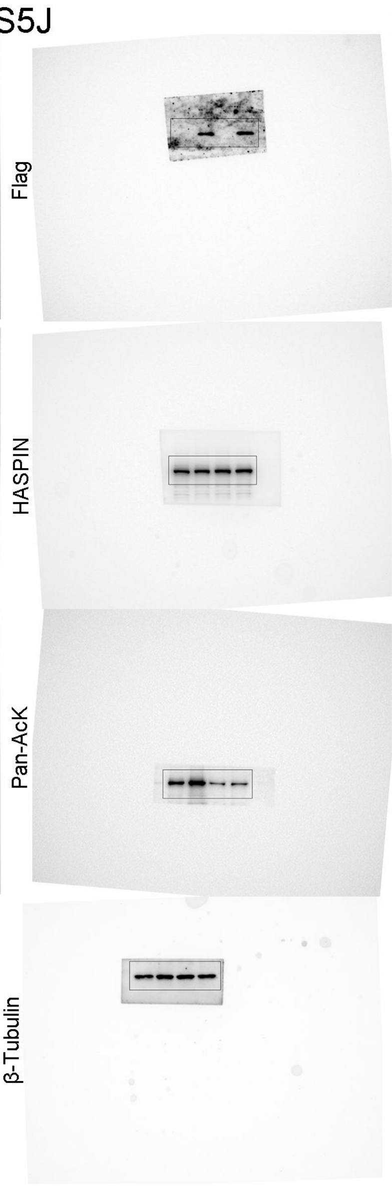

S5K

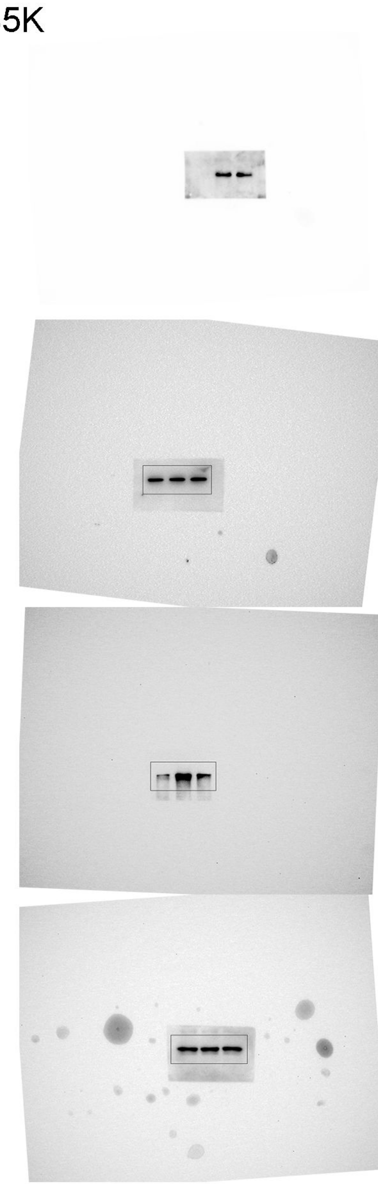

Supplement: Supplementary file 10 — Original Western Blots [file 41419_2026_8658_MOESM10_ESM.pdf]
